# Shared parameter model for competing risks and different data summaries in meta‐analysis: Implications for common and rare outcomes

**DOI:** 10.1002/jrsm.1371

**Published:** 2019-08-22

**Authors:** Howard Thom, José A. López‐López, Nicky J. Welton

**Affiliations:** ^1^ Bristol Medical School: Population Health Sciences University of Bristol Bristol UK; ^2^ Department of Basic Psychology & Methodology, Faculty of Psychology University of Murcia Murcia Spain

**Keywords:** competing risks, network meta‐analysis, meta‐analysis, shared parameter models, different data summaries, rare events

## Abstract

This paper considers the problem in aggregate data meta‐analysis of studies reporting multiple competing binary outcomes and of studies using different summary formats for those outcomes. For example, some may report numbers of patients with at least one of each outcome while others may report the total number of such outcomes. We develop a shared parameter model on hazard ratio scale accounting for different data summaries and competing risks. We adapt theoretical arguments from the literature to demonstrate that the models are equivalent if events are rare. We use constructed data examples and a simulation study to find an event rate threshold of approximately 0.2 above which competing risks and different data summaries may bias results if no adjustments are made. Below this threshold, simpler models may be sufficient. We recommend analysts to consider the absolute event rates and only use a simple model ignoring data types and competing risks if all of underlying events are rare (below our threshold of approximately 0.2). If one or more of the absolute event rates approaches or exceeds our informal threshold, it may be necessary to account for data types and competing risks through a shared parameter model in order to avoid biased estimates.

## 1 INTRODUCTION

Aggregate data meta‐analysis synthesizes the results on outcomes that have been reported by studies such as randomized controlled trials (RCTs). In meta‐analysis of binary outcomes, a common complication is that RCTs report more than one outcome and that these outcomes are correlated or indeed competing; for instance, an RCT may censor patients who experience a stroke, and thus, a later myocardial infarction will not be reported.^1^, ^2^ A further complication is that some studies may report different aggregate summary format of the outcomes of interest; for example, some may report numbers of patients with at least one of each outcome, while others report the total number of each outcome, counting patients more than once. These issues of competing risks and differently reported data are also present in network meta‐analysis (NMA) that aims to compare multiple interventions via connected networks of evidence.^3^

It is known from the statistical literature that competing risks have limited impact if events are rare, and scenarios have been identified where it is not necessary to account for competing risks in meta‐analysis.^4^, ^5^ However, if events are not rare, then not accounting for these complications can affect treatment comparisons.^6^ Differences in conclusions of meta‐analyses and NMA are important as they are the recommended approach for treatment effect estimation and indirect comparison by health care authorities (eg, National Institute of Healthcare and Decision Making in the United Kingdom) and international medical decision‐making societies (eg, International Society for Pharmacoeconomics and Outcomes Research and Society for Medical Decision Making).^7^, ^8^ Despite the importance, meta‐analysis and NMA on outcomes that are not rare commonly use simple analyses that may be biased. Examples can be found in depression,^9^, ^10^ relapsing remitting multiple sclerosis,^11^ and 5‐year survival and mortality rates in a range of cancers.^12^ Furthermore, health economic decision models may require treatment effects on multiple outcomes, requiring that the interdependence be explicitly modelled.^13^ The bias in results from ignoring outcome interdependence can be magnified if used in such decision models.^14^

Several approaches have been proposed to account for competing risks in meta‐analysis and NMA, including modelling functional relationships between outcomes,^1^ linear regression of outcomes of interest on surrogate outcomes,^15^, ^16^ and normal approximations.^17^ If individual patient data are available from the RCTs, within‐study correlation between outcomes in a meta‐analysis can be explicitly modelled,^18^ but it is rare for such individual patient data to be available for all RCTs. Meta‐analysis of the cumulative incidence function, the expected proportion of cause‐specific events over time, has also been considered.^19^

There has been less work developing methods to combine differently summarized data in (network) meta‐analysis with event outcomes. Models with different treatment effects for each data summary but related by a common random effect have been proposed.^20^ Due to its greater flexibility and explicit modelling of underlying relationships, we adopt the approach of shared parameter models.^3^, ^21^, ^22^ These have been used to combine binomial data with estimated log odds ratios or mean survival.^23^ Illustrating their flexibility, shared parameter models have also been used to combine continuous, categorical, and binary data.^24^

In this paper, we develop a novel shared parameter competing risk model to simultaneously account for all these complications in a NMA. This extends earlier work on competing risks and modelling of functions of shared parameters.^1^, ^25^ Our work is motivated by a NMA comparing anticoagulants for the prevention of stroke in atrial fibrillation. We investigate theoretical reasons why the adjustments are not necessary when events are rare and use constructed data to identify scenarios where results can be substantially biased if an unadjusted model is used. We also present results from a simple simulation study exploring the conditions under which adjusted and unadjusted analyses can give biased results.

## 2 MOTIVATING EXAMPLE: DIRECTLY ACTING ORAL ANTICOAGULANTS FOR STROKE PREVENTION IN ATRIAL FIBRILLATION

The objective of our case study was to compare first‐line treatments for the prevention of stroke, myocardial infarction (MI), bleed, and death in atrial fibrillation. Our case study is based on a systematic literature review and Bayesian NMA comparing treatments for the prevention of stroke in atrial fibrillation (AF), which has been described previously in the literature.^26^, ^27^ AF is the most common cardiac arrhythmia and substantially increases the risk of thromboembolic stroke due to blood pooling in the left atrium and systemic embolization to the brain.^28^ Treatments for prevention of stroke in AF include the anticoagulant warfarin as well as the more recently developed directly acting (or nonvitamin K antagonist) oral anticoagulants (DOACs).^29^, ^30^ However, all anticoagulants carry the risk of internal bleeding.^31^ Our NMA compared warfarin (international normalized ratio (INR) target range 2‐3) with the DOACs apixaban (twice daily 5 mg), dabigatran (twice daily 150 mg), edoxaban (once daily 60 mg), and rivaroxaban (once daily 20 mg).

The four outcomes of interest were ischaemic stroke, MI, clinically relevant bleeding, and death. The systematic literature review identified 20 RCTs comparing DOACs to warfarin and reporting on these outcomes.^26^, ^27^ However, an additional thirteen outcomes were reported by these studies: bleeding (distinct from clinically relevant bleeding), minor bleeding, fatal bleeding, transient ischaemic attack (TIA), fatal stroke, composite clinically relevant bleeding (again distinct from clinically relevant bleeding), hospital admission, death (cardiovascular specific), arterial event, pulmonary embolism, extracranial minor bleeding, systemic embolism (SE), and intracranial bleeding (ICH) (to which we added haemorrhagic stroke, under clinical advice).

A further complication was that the data from the RCTs were summarized in three different formats, which are described in Table 1. Data format 1 summarizes only the first event for each individual, after which they were assumed censored; this data format involves competing risks as patients reporting one outcome are censored and cannot report further outcomes. Data format 2 summarizes only the total number of individuals experiencing at least one of each outcome. In this second form of summary, each individual may count towards more than one outcome, but only once for each outcome. Data format 3 gives the total number of events of each outcome across all patients. Data formats 2 and 3 do not involve competing risks. Five studies reported data format 1, 13 reported format 2, and two reported format 3. Although our interest is only on the four key outcomes of ischaemic stroke, MI, clinically relevant bleeding, and death, as these are competing risks, the additional 13 outcomes must also be modelled. Further details on the data are included in the Supporting Information.

**Table 1:** Different data formats and models included in the network meta‐analysis

Data Format Index in Model	Data Format	Competing risksRisks?	Number of studiesStudies	Likelihood and Link Function in Shared Parameter Model
*j* = 1	Reports only first event for each patient, after which they are censored	Yes	5	Poisson for total number of events, multinomial for each outcome with log link
*j* = 2	Total number with at least one of each outcome	No	13	Binomial likelihood with complementary log log link. Poisson with log for death
*j* = 3	Total number of events across patients	No	2	Poisson for total number of events with log link

## 3 METHODS

We first denote the number of studies in our analysis *N*_*s*_ and number of arms *K*_*i*_ in trial *i* = 1,…,*N*_*s*_. Let $r_{\mathrm{ikm}}^{\left(j\right)}$ be the number of events in study *i*, arm *k* = 1,…,*K*_*i*_ (with data format *j* = 1,2,3), and outcome *m* (*m* = 1,…,*M*). In our application, the final event *M* represents mortality. The treatment *t*_*ik*_ and number randomized *n*_*ik*_ are independent of outcome *m*. The total patient years at risk or exposure time, which is also the same for every outcome, is denoted *E*_*ik*_, while mean patient follow‐up time is ${\bar{T}}_{\mathrm{ik}}=\frac{E_{\mathrm{ik}}}{n_{\mathrm{ik}}}$. Not every study necessarily reports each outcome.

### 3.1 Simple odds ratio model: separate NMA models for each outcome, ignoring data type

The simplest approach is to model each outcome separately (ignoring the competing risk nature of the outcomes), to treat all the studies as estimating the same probability of outcome (regardless of data format), and to ignore the follow‐up time or exposure. In this case, a binomial likelihood is given independently for each outcome, with the number randomized, *n*_*ik*_, as the denominator.

The likelihood for each outcome *m* reported in arm *k* of study *i* is

$$r_{\mathrm{ikm}}^{\left(j\right)}\sim \mathrm{Bin}\left(p_{\mathrm{ikm}},n_{\mathrm{ik}}\right),\text{for each data format type}\;j=1,2,3.$$

This is the number of events $r_{\mathrm{ikm}}^{\left(j\right)}$ of outcome *m* which occurred in the *n*_*ik*_ patients in arm *k* of trial *i*. We then use a fixed‐effect NMA model assuming consistency of effects on the log odds scale, so that the relative treatment effects are log odds ratios^3^

$$\text{logit}\left(p_{\mathrm{ikm}}\right)=\mu_{\mathrm{im}}+d_{t_{\mathrm{ik}}m}-d_{t_{i1}m}$$

where the baseline log odds *μ*_*im*_ of outcome *m* are treated as nuisance parameters and vague priors are placed on them

$$\mu_{\mathrm{im}}\sim N\left(0,0.0001\right)$$

and vague priors are also placed on the log odds ratios *d*_*tm*_ for all outcomes *m* and treatments *t*

$$d_{\mathrm{tm}}\sim N\left(0,0.0001\right)\;\text{for}\;t\neq 1$$

$$d_{1m}=0$$

Vague priors may introduce numerical issues with convergence; if this occurs, less diffuse priors can be explored.

Preliminary investigations found that a fixed rather than random treatment effect model was adequate for the AF application. The key implicit assumption of this model for our purposes is that *r*_*ikm*_ is the number of patients who have at least one event of outcome *m* and that patients who have multiple such events are only counted once. The interpretation of *d*_*tm*_ is therefore of a log odds ratio of the event regardless of whether it is the first or a subsequent event. We now describe alternative models that do not make this assumption.

### 3.2 Shared parameter model on hazard ratios accounting for competing risks and different data summaries

The first modification is to put the model on the log hazard ratio rather than log odds ratio scale, in order to estimate a single model incorporating all the different data types. In our shared parameter models, *λ*_*ikm*_ is the hazard of outcome *m* in arm *k* of study *i*. Although the interpretation of the number of events $r_{\mathrm{ikm}}^{\left(j\right)}$ is different for each of the data type *j*, the interpretation of the *λ*_*ikm*_ is always the same. The NMA model is put on the log hazard scale

(1) $$\log\left(\lambda_{\mathrm{ikm}}\right)=\omega_{\mathrm{im}}+h_{t_{\mathrm{ik}}m}-h_{t_{i1}m}$$

where the baseline log hazards *ω*_*im*_ are treated as nuisance parameters and priors are placed on them

$$\omega_{\mathrm{im}}\sim N\left(0,0.5\right)$$

with the precision 0.5 corresponding to a standard deviation of approximately 1.41. The log hazard ratios, *h*_*tm*_, for treatment *t* relative to treatment 1 for outcome *m* are given the same priors

$$h_{\mathrm{tm}}\sim N\left(0,0.5\right)\;\text{for}\;t\neq 1$$

$$h_{1m}=0$$

More vague priors did not converge with our atrial fibrillation example but are explored in simulation below. We recommend exploring more diffuse priors in other applications of the model.

A different likelihood and link function is used for each of the three data formats *j*, but all contribute evidence to the *λ*_*ikm*_ and thus to the treatment effects *h*_*tm*_.

#### 3.2.1 Studies reporting number of patients whose first event is of a given type: data type *j* = 1

In studies of this type, only the first event is recorded for each individual, and they are assumed censored from the time at which that first event occurs. The outcomes are therefore competing risks and must be modelled jointly. This type of data are labelled *j* = 1, and the $r_{\mathrm{ikm}}^{\left(1\right)}$ are the number of individuals with first event of type *m*. We label $R_{\mathrm{ik}}=\sum_{m=1}^{M}r_{\mathrm{ikm}}^{\left(1\right)}$ the total number of patients having an event of any type in arm *k* of study *i*. Recalling that *E*_*ik*_ is the observed person years at risk, our model for this data uses a Poisson and multinomial likelihood

$$R_{\mathrm{ik}}\sim \text{Poisson}\left(E_{\mathrm{ik}}\sum_{m=1}^{M}\lambda_{\mathrm{ikm}}\right)$$

$$\left(r_{\mathrm{ik}1}^{\left(1\right)},r_{\mathrm{ik}2}^{\left(1\right)},\ldots ,r_{\mathrm{ikM}}^{\left(1\right)}\right)\sim \text{Multinomial}\left(\left(\frac{\lambda_{\mathrm{ik}1}}{\sum_{m=1}^{M}\lambda_{\mathrm{ikm}}},\ldots ,\frac{\lambda_{\mathrm{ikM}}}{\sum_{m=1}^{M}\lambda_{\mathrm{ikm}}}\right);R_{\mathrm{ik}}\right)$$

The hazards *λ*_*ikm*_ are then modelled on the log scale via Equation (1).

#### 3.2.2 Studies reporting number of patients experiencing at least one event for each type: data type *j* = 2

In this type of study, each patient may count towards more than one event type, but only once for each event type. There are therefore no competing risks to consider. This data are labelled *j* = 2, and $r_{\mathrm{ikm}}^{\left(2\right)}$ are here the number of patients with at least one event of type *m*. As mortality can only occur once, the number of mortalities $r_{\mathrm{ikM}}^{\left(2\right)}$ must be modelled differently to other events. Assuming an average follow‐up time ${\bar{T}}_{\mathrm{ik}}$ for each patient, the likelihood for nonmortality events is

$$r_{\mathrm{ikm}}^{\left(2\right)}\sim \text{binomial}\left(p_{\mathrm{ikm}},n_{\mathrm{ik}}\right)$$

This is the same as in the simple model, but *p*_*ikm*_ is now the probability that an individual has one or more events of type *i* over the mean follow‐up ${\bar{T}}_{\mathrm{ik}}$. The appropriate link function is therefore the complementary log‐log

$$\text{cloglog}\left(p_{\mathrm{ikm}}\right)=\log\left({\bar{T}}_{\mathrm{ik}}\right)+\log\left(\lambda_{\mathrm{ikm}}\right)$$

The likelihood for the number of mortalities is

$$r_{\mathrm{ikM}}^{\left(2\right)}\sim \text{Poisson}\left(E_{\mathrm{ik}}\lambda_{\mathrm{ikM}}\right)$$

The hazards *λ*_*ikm*_ are again linked to the log hazard ratios via Equation (1).

#### 3.2.3 Studies reporting the total number of events of each type: data type *j* = 3

In this final type of study, data are labelled *j* = 3, and $r_{\mathrm{ikm}}^{\left(3\right)}$ are the total number of events of type *m*, including repeat events with patients, for given person years at risk *E*_*ik*_. The likelihood is simply

$$r_{\mathrm{ikm}}^{\left(3\right)}\sim \text{Poisson}\left(E_{\mathrm{ik}}\lambda_{\mathrm{ikm}}\right)$$

with *λ*_*ikm*_ modelled by Equation (1). This completes our specification of a shared parameter model accounting for the three types of study data and for competing risks when they are present.

### 3.3 Equivalence of simple and shared parameter models if events are rare: asymptotic arguments

We have so far developed two models. Section 3.1 developed a simple odds ratio model that assumes all outcomes are independent and models the three data summary types in the same way. Section 3.2 developed a shared parameter hazard ratio model that uses different models for the three data types *j* = 1,2,3. In the present section, we give theoretical demonstrations that if events are rare, the models for these three data summaries are equivalent. As odds and hazard ratio models are equivalent if events are rare, the hazard ratio model for studies reporting number of patients with at least one of each event type described in Section 3.2.2 (data type *j* = 2) is equivalent to the simple odds ratio model of Section 3.1.^32^ The demonstrations below therefore establish that the simple and shared parameter models give equivalent results if events are rare. Note that the equivalence of competing and noncompeting risk NMA is further established in the Supporting Information, but our argument below does not rely on this result.

#### 3.3.1 Equivalence of models for types 1 and 3 under rare events

Recall that data of type 1 were number of patients whose first event was of a given type. Total number of events $R_{\mathrm{ik}}=\sum_{m=1}^{M}r_{\mathrm{ikm}}^{\left(1\right)}$ was modelled using a Poisson distribution with parameter $E_{\mathrm{ik}}\sum_{i=1}^{M}\lambda_{\mathrm{ikk}}$ while the number of first events of each type $\left(r_{\mathrm{ik}1}^{\left(1\right)},r_{\mathrm{ik}2}^{\left(1\right)},\ldots ,r_{\mathrm{ikM}}^{\left(1\right)}\right)$ followed a multinomial distribution. Dropping the indices for arm *k* and study *i* the likelihood is proportional to

$$L_{1}\left(r_{1}^{\left(1\right)},\ldots ,r_{M}^{\left(1\right)}\right)\propto \prod_{m=1}^{M}{\left(\frac{\lambda_{m}}{\sum \lambda_{l}}\right)}^{r_{m}^{\left(1\right)}}E^{R}e^{-E\sum \lambda_{l}}$$

Noting $R=\sum_{m=1}^{M}r_{m}^{\left(1\right)}$ gives

$$L_{1}\left(r_{1}^{\left(1\right)},\ldots ,r_{M}^{\left(1\right)}\right)\propto \prod_{m=1}^{M}\left(\lambda_{m}^{r_{m}^{\left(1\right)}}\right){\left(\frac{1}{\sum \lambda_{l}}\right)}^{R}E^{R}e^{-E\sum \lambda_{l}}$$

Data of type 3 are the total number of each event, so the likelihood is a product of *M* Poisson distributions with parameters *Eλ*_*m*_. Removing constants, this becomes

$$L_{3}\left(r_{1}^{\left(3\right)},\ldots ,r_{M}^{\left(3\right)}\right)\propto \prod_{m=1}^{M}\left(\lambda_{m}^{r_{m}^{\left(1\right)}}\right)e^{-E\sum \lambda_{l}}$$

*L*_1_ and *L*_3_ are always different by a, parameter dependent and therefore nonconstant, factor of ${\left(\frac{1}{\sum \lambda_{l}}\right)}^{R}$, but this will be minimized when *R* (the total number of events) is small, which is when events are rare.

#### 3.3.2 Equivalence of models for types 2 and 3 under rare events

In this case, we only need to consider nonmortality events as the numbers of mortalities for type 2 and 3 data are both modelled as Poisson likelihoods with parameter *E*_*ik*_*λ*_*ikM*_. As events are assumed independent under both models, we consider only one event *m*. Recall the type 2 likelihood

$$r_{\mathrm{ikm}}^{\left(2\right)}\sim \text{binomial}\left(p_{\mathrm{ikm}},n_{\mathrm{ik}}\right)$$

(2) $$\text{cloglog}\left(p_{\mathrm{ikm}}\right)=\log\left({\bar{T}}_{\mathrm{ik}}\right)+\log\left(\lambda_{\mathrm{ikm}}\right)$$

Following the law of rare events,^33^ this binomial distribution can be approximated by a Poisson if *n*_*ik*_ is large and *p*_*ikm*_ is small (ie, rare events)

(3) $$r_{\mathrm{ikm}}^{\left(2\right)}\sim \text{Poisson}\left(p_{\mathrm{ikm}}n_{\mathrm{ik}}\right)$$

Noting the relation between mean follow‐up and exposure time ${\bar{T}}_{\mathrm{ik}}=\frac{E_{\mathrm{ik}}}{n_{\mathrm{ik}}}$, Equation (2) gives a further expression for the event probability

pikm=1−eλikmEiknik

If the rate *λ*_*ikm*_ is low, the exponential can be approximated using $\lim_{x\to 0}e^{x}=1-x$, and substituting for *p*_*ikm*_ in the Poisson likelihood of Equation (3) gives

$$r_{\mathrm{ikm}}^{\left(2\right)}\sim \text{Poisson}\left(E_{\mathrm{ik}}\lambda_{\mathrm{ikm}}\right)$$

This is exactly the likelihood for data of type 3.

This completes our demonstration that the models for the three data types are equivalent and therefore that the shared parameter hazard ratio model is equivalent to the simple odds ratio model, if events are rare.

### 3.4 The impact on results if events are more common: constructed data

We explored the conditions under which the simple and shared parameter models will give different results. This was done by constructing AF datasets with increased rates of events. We increased the number of events of each outcome, while keeping the number of patients constant, in each RCT by factors of 1 (base case), 2, 5, 10, and 20. We capped the number of events of each type at the number of patients. These five constructed datasets were then analyzed using both the simple odds ratio model and the shared parameter hazard ratio model.

### 3.5 Simulation study

As the AF example was a single replicate, we conducted a simulation study to compare the NMA results of the simple odds ratio and shared parameter models on a variety of simulated sets of trial results. Best practice guidelines for simulation studies were followed.^34^ We simulated datasets from the parametric shared parameter model to (1) explore the performance of estimating a shared parameter model and (2) identify conditions where the simple odds ratio approach performed adequately.

All our scenarios assume that there are three treatments being studied on two outcomes over 10 RCTs. We do not specify if outcomes 1 or 2 are positive (eg, recovery) or negative (eg, stroke), but for simplicity, we specify neither are death to avoid issues of censoring. Simulated studies were all two‐arm RCTs with 100 patients on each arm and a follow‐up of 1 year. Simulated results of the RCTs were modelled to be in one of the three formats of the AF example. Full details of the data formats and included treatments are in Table 2. Treatment 1 was modelled as the reference treatment. Log hazard ratios of treatment 2 relative to treatment 1 were −0.25 and 0.25 on outcomes 1 and 2, respectively (in Equation (1), these are denoted *h*_2*m*_ for *m* = 1,2). Log hazard ratios of treatment 3 relative to treatment 1 were 0.25 and −0.25 on outcomes 1 and 2, respectively (*h*_3*m*_ for *m* = 1,2). The log hazards on treatment 1 in each trial (denoted *ω*_*im*_ for *i* = 1,…,10) were set such that the average event rate for each outcome (*λ*_*ikm*_ for trial *i*, treatment *k* = 1,2,3, outcome *m*) was 0.05, 0.1, 0.2, 0.3, and 0.4. This set of five event rates for each outcome gives 25 scenarios in total. Numbers of events of each outcome on each arm were simulated randomly, and the results fed into the simple odds ratio and shared parameter models.

**Table 2:** Studies included in simulation study

Study	Data Format	Number of Patients on Treatment 1	Number of Patients on Treatment 2	Number of Patients on Treatment 3
1	1	100	100	0
2	1	100	0	100
3	1	100	100	0
4	1	0	100	100
5	2	100	100	0
6	2	100	0	100
7	2	100	0	100
8	3	0	100	100
9	3	100	0	100
10	3	100	100	0

*Notes.* All are two‐arm RCTs with follow‐up of 1 year.

The targets of estimation by the models were the *h*_*km*_ for treatments *k* = 2 and *k* = 3 on both outcomes, and the performance of each model was assessed over estimates ${\hat{h}}_{\mathrm{km}\left(l\right)}$ on each simulation *l* = 1,…,*n*_*sim*_. We prespecified that the bias ( $\frac{1}{n_{\mathrm{sim}}}\sum_{k=1}^{2}\sum_{l=1}^{n_{\mathrm{sim}}}{\hat{h}}_{\mathrm{km}\left(l\right)}-h_{\mathrm{km}}$ *)* and coverage (probability that ${\hat{h}}_{\mathrm{km}\left(l\right)}^{\mathrm{low}}\leq h_{\mathrm{km}}\leq {\hat{h}}_{\mathrm{km}\left(l\right)}^{\text{high}}$ for all *l* and *k*) would be the performance measures. Number of simulations *n*_*sim*_ was calculated to be 2500 so that the mean squared error of the coverage was less than 0.01 and for bias was 0.02.^34^

To explore the impact of priors in the shared parameter model when events are rare, we repeated the simulation study for event rates 0.05 and 0.1, and again with *n*_*sim*_ = 2500, but using baseline log hazard prior for outcome *m* of study *i*

$$\omega_{\mathrm{im}}\sim N\left(0,0.05\right)$$

and log hazard ratio prior, for treatment *t* relative to treatment 1

*h*_*tm*_~*N*(0,0.05) for *t* ≠ 1

These correspond to standard deviations of approximately 4.47. More vague priors were not found to reliably converge for the simulation study data.

### 3.6 Model implementation

The NMA models presented were implemented in OpenBUGS version 3.2.3 rev 1012.^35^ We used two chains with 60 000 iterations for burn‐in and 30 000 iterations for posterior sampling in the AF base case and constructed data examples. The simulation study, due to greater computational resources required, used two chains but with 30 000 iterations for burn‐in and 10 000 iterations for sampling. Convergence was assessed using Brooks‐Gelman‐Rubin (GBR) statistics and visual inspection of the history plots.^36^ Data were cleaned and saved in the appropriate BUGS format in the R programming language version 3.1.2.^37^ The inflation of event rates, summarizing of results, and generation of figures necessary for the constructed data examples were conducted in R, as was the simulation study. Code for the novel shared parameter model is presented in the Supporting Information while code for simulation study is available on request.

## 4 RESULTS

### 4.1 Comparison of model results

The results of the two models are presented in Table 3. The “simple OR” results correspond to the simple model on odds ratios while the “shared parameter HR” results correspond to the hazard ratios estimated by the shared parameter model. These odds and hazard ratios are relative to the reference of warfarin (INR 2‐3). Odds ratios or hazard ratios greater than 1 suggest worse outcomes (eg, higher odds or hazard of stroke) on the DOAC compared with warfarin. If the 95% credible intervals include 1, they suggests that we cannot rule out no difference in treatment effect. Comparing across DOACs, higher odds or hazard ratios again indicate worse outcomes while overlapping 95% credible intervals suggest uncertainty about which performs better. Comparison of treatments has been described and interpreted in detail in an earlier publication.^26^ Broadly, apixaban performs well (lower odds and hazards) across all outcomes of stroke, MI, death, and bleed. Dabigatran has the lowest odds and hazards on stroke but elevated danger of MI and bleed. All DOACs have lower odds/hazards of death and perform similarly to each other.

**Table 3:** Comparison of simple odds ratio and shared parameter model results, odds ratios, and hazard ratios are relative to warfarin with 95% credible intervals between brackets

	Stroke Shared Parameter HR	Stroke Simple OR	MI Shared Parameter HR	MI Simple OR	Death Shared Parameter HR	Death Simple OR	Bleed Shared Parameter HR	Bleed Simple OR
Apixaban	0.9 (0.73, 1.1)	0.91 (0.73, 1.1)	0.83 (0.65, 1.1)	0.87 (0.67, 1.10)	0.89 (0.80, 0.99)	0.88 (0.79, 0.99)	0.81 (0.69, 0.95)	0.81 (0.70, 0.95)
Dabigatran	0.75 (0.58, 0.97)	0.76 (0.58, 0.98)	1.20 (0.93, 1.70)	1.30 (0.95, 1.70)	0.89 (0.78, 1.00)	0.88 (0.77, 1.00)	1.10 (0.92, 1.20)	1.10 (0.92, 1.30)
Edoxaban	1.00 (0.83, 1.20)	1.00 (0.84, 1.20)	0.94 (0.74, 1.20)	0.96 (0.76, 1.2)	0.91 (0.83, 1.00)	0.91 (0.82, 1.00)	0.88 (0.82, 0.94)	0.85 (0.78, 0.92)
Rivaroxaban	0.92 (0.73, 1.10)	0.93 (0.74, 1.20)	0.79 (0.61, 1.00)	0.8 (0.61, 1.00)	0.82 (0.69, 0.99)	0.83 (0.69, 1.00)	1.00 (0.98, 1.10)	1.10 (0.98, 1.20)

Abbreviations: HR, hazard ratio; OR, odds ratio; MI, myocardial infarction.

The methodologically interesting finding is that the results of the simple and shared parameter models are very similar. The point estimates and the limits of the 95% credible intervals are either very similar or identical. The greatest divergence between the models is in MI for dabigatran, perhaps because this is the least rare event, but even in this case, the difference in results is small.

### 4.2 Results of constructed data examples

**Figure 1:**
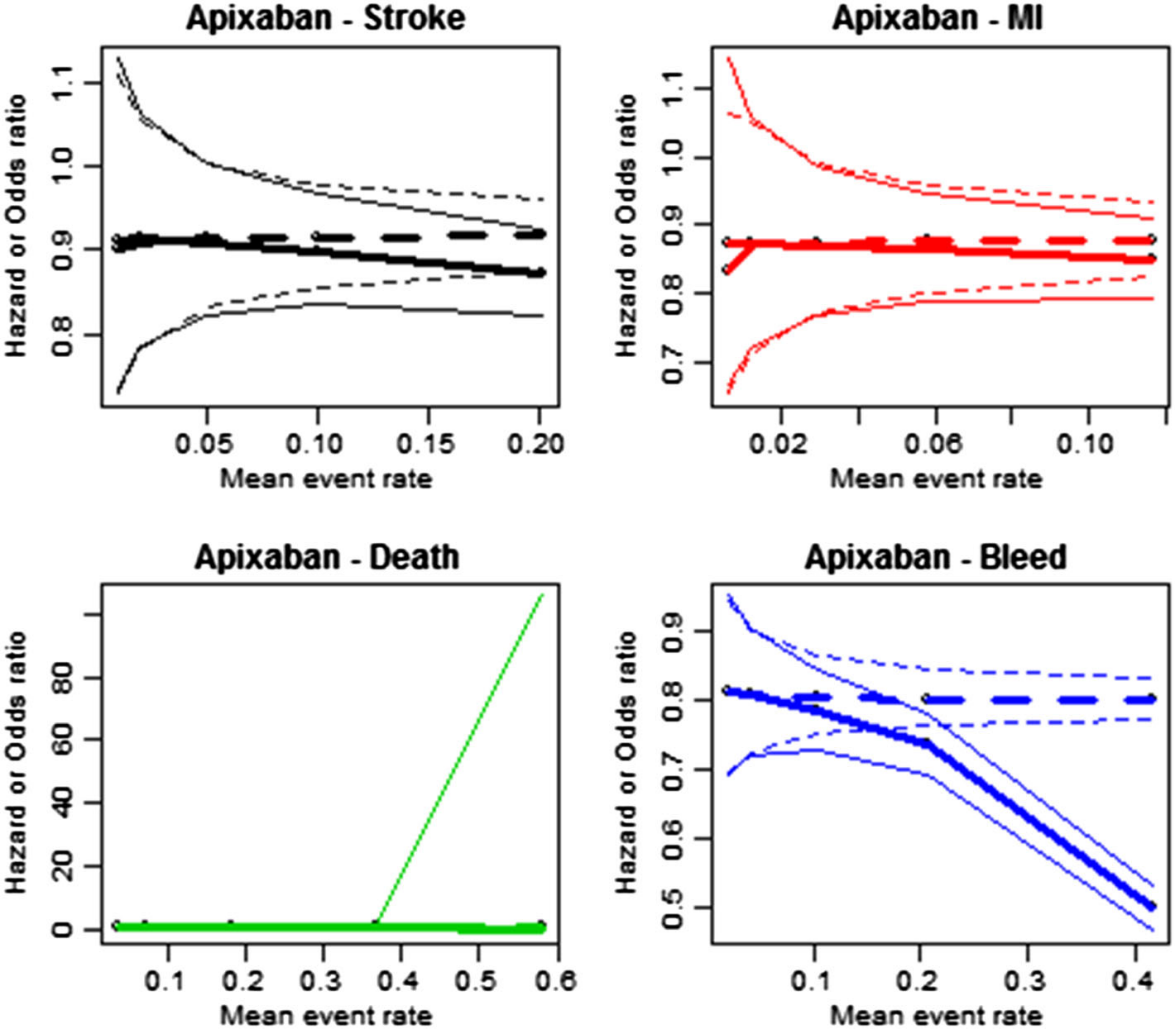
Comparison of estimated adjusted HR (dotted) and simple OR (solid) for apixaban under constructed data scenarios. Thin lines represent upper and lower 95% credible interval limits

**Figure 2:**
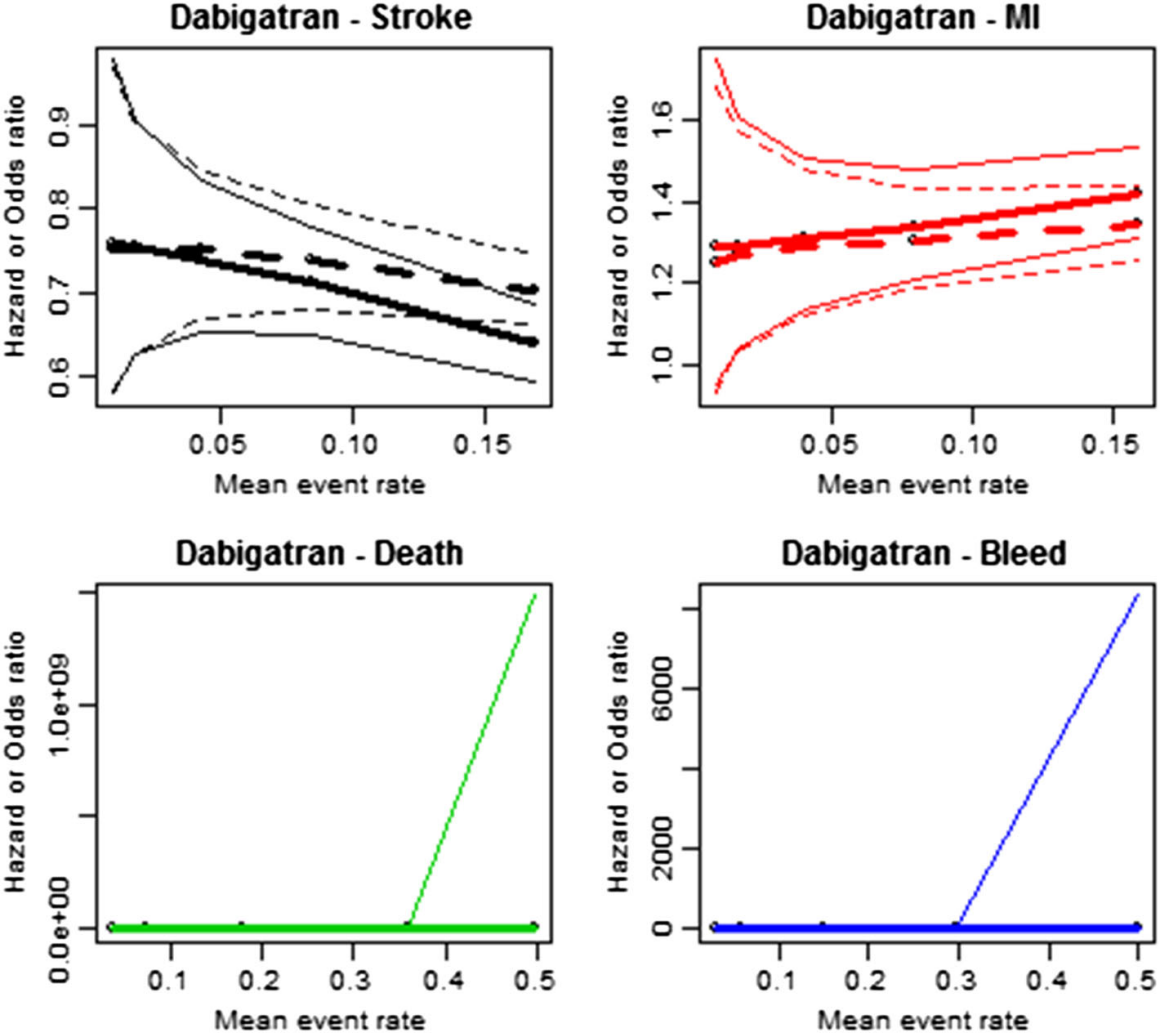
Comparison of estimated adjusted HR (dotted) and simple OR (solid) for dabigatran under constructed data scenarios. Thin lines represent upper and lower 95% credible interval limits

Mean and 95% credible intervals for the odds and hazard ratios estimated by the two models under the increased event rate scenarios are summarized for apixaban and dabigatran in Tables 4 and 5, respectively. Results for rivaroxaban and edoxaban are presented in the Supporting Information. Under the base case, the event rates are sufficiently low that the two models agree in their point and uncertainty estimates. However, as the rates are gradually increased, the point estimates and credible intervals become more different. This divergence in results is illustrated in Figures 1 and 2. It appears that as the event rate exceeds 0.2, the credible intervals no longer overlap and conclusions change substantially.

**Table 4:** Comparison of constructed data results for apixaban vs warfarin

Scenario	Outcome	Adjusted HR^a^ Mean (95% CrI)	Simple OR^b^ Mean (95% CrI)	Event Rate^c^
Base rate	Ischaemic stroke	0.901 (0.729, 1.11)	0.913 (0.734, 1.13)	0.0101
	MI	0.832 (0.655, 1.06)	0.874 (0.666, 1.14)	0.00584
	Death (all causes)	0.887 (0.798, 0.986)	0.883 (0.792, 0.986)	0.0366
	Clinically relevant bleeding	0.811 (0.693, 0.945)	0.812 (0.696, 0.95)	0.0207
Inflate by 2	Ischaemic stroke	0.909 (0.783, 1.06)	0.913 (0.784, 1.07)	0.0202
	MI	0.864 (0.711, 1.05)	0.874 (0.72, 1.06)	0.0117
	Death (all causes)	0.893 (0.827, 0.963)	0.876 (0.809, 0.949)	0.0732
	Clinically relevant bleeding	0.807 (0.722, 0.901)	0.807 (0.721, 0.902)	0.0415
Inflate by 5	Ischaemic stroke	0.915 (0.832, 1.01)	0.909 (0.822, 1)	0.0505
	MI	0.873 (0.771, 0.99)	0.87 (0.767, 0.986)	0.0292
	Death (all causes)	0.896 (0.853, 0.941)	0.847 (0.798, 0.9)	0.183
	Clinically relevant bleeding	0.804 (0.751, 0.862)	0.785 (0.728, 0.844)	0.104
Inflate by 10	Ischaemic stroke	0.915 (0.855, 0.979)	0.899 (0.834, 0.968)	0.101
	MI	0.876 (0.802, 0.958)	0.864 (0.788, 0.946)	0.0584
	Death (all causes)	0.897 (0.866, 0.928)	0.697 (0.654, 0.743)	0.366
	Clinically relevant bleeding	0.801 (0.762, 0.842)	0.734 (0.692, 0.78)	0.207
Inflate by 20	Ischaemic stroke	0.917 (0.874, 0.961)	0.871 (0.821, 0.925)	0.202
	MI	0.877 (0.823, 0.934)	0.848 (0.791, 0.91)	0.117
	Death (all causes)	0.999 (0.97, 1.03)	0.0429 (4.33e‐06, 106)^d^	0.581
	Clinically relevant bleeding	0.801 (0.773, 0.83)	0.499 (0.469, 0.532)	0.415

*Notes.* Results are shaded if the 95% credible intervals do not overlap.

a Adjusted HR is the hazard ratio estimated by the model accounting for competing risks and differently reported data.

b Simple OR is the odds ratio estimated by the model that disregards competing risks and differently reported data.

c Averaged over all arms and all trials.

d Anomalous results due to high event rate.

**Table 5:** Comparison of constructed data results for dabigatran vs warfarin

Scenario	Outcome	Adjusted HR^a^ Mean (95% CrI)	Simple OR^b^ Mean (95% CrI)	Event Rate^c^
Base rate	Ischaemic stroke	0.751 (0.583, 0.969)	0.757 (0.583, 0.978)	0.00845
	MI	1.25 (0.932, 1.68)	1.29 (0.949, 1.75)	0.00796
	Death (all causes)	0.886 (0.78, 1.01)	0.882 (0.772, 1.01)	0.0359
	Clinically relevant bleeding	1.07 (0.916, 1.24)	1.08 (0.925, 1.26)	0.0299
Inflate by 2	Ischaemic stroke	0.752 (0.627, 0.904)	0.754 (0.628, 0.907)	0.0169
	MI	1.27 (1.03, 1.57)	1.29 (1.04, 1.61)	0.0159
	Death (all causes)	0.882 (0.807, 0.968)	0.873 (0.79, 0.964)	0.0719
	Clinically relevant bleeding	1.07 (0.966, 1.19)	1.09 (0.973, 1.22)	0.0597
Inflate by 5	Ischaemic stroke	0.753 (0.67, 0.846)	0.74 (0.655, 0.835)	0.0423
	MI	1.29 (1.12, 1.48)	1.31 (1.14, 1.51)	0.0398
	Death (all causes)	0.863 (0.814, 0.915)	0.831 (0.772, 0.893)	0.18
	Clinically relevant bleeding	1.08 (1.01, 1.15)	1.11 (1.03, 1.2)	0.149
Inflate by 10	Ischaemic stroke	0.739 (0.68, 0.803)	0.713 (0.652, 0.78)	0.0845
	MI	1.31 (1.19, 1.43)	1.34 (1.21, 1.48)	0.0796
	Death (all causes)	0.771 (0.737, 0.806)	0.611 (0.561, 0.665)	0.359
	Clinically relevant bleeding	1.08 (1.03, 1.13)	1.2 (1.11, 1.29)	0.299
Inflate by 20	Ischaemic stroke	0.703 (0.662, 0.745)	0.639 (0.594, 0.688)	0.169
	MI	1.34 (1.26, 1.44)	1.41 (1.31, 1.53)	0.159
	Death (all causes)	1.89 (0.285, 19.2)	86.1 (0.0001, 1.49e+09)^d^	0.499
	Clinically relevant bleeding	1 (0.966, 1.04)	8.5 (0.00728, 8334)^d^	0.5

*Notes.* Results are shaded if the 95% credible intervals do not overlap.

a Adjusted HR is the hazard ratio estimated by the model accounting for competing risks and differently reported data.

b Simple OR is the odds ratio estimated by the model that disregards competing risks and differently reported data.

c Averaged over all arms and all trials.

d Anomalous results due to high event rate.

### 4.3 Simulation study results

The bias results on outcomes 1 and 2 are presented in Tables 6 and 7, respectively, while coverage results on outcomes 1 and 2 are presented in Tables 8 and 9, respectively. In general, the shared parameter model has higher bias and lower coverage on each outcome than the simple odds ratio model when events are rare, but this reverses as event rates of the outcome being studied increase. Limited data and influence of the prior distributions are a likely explanation for the superior performance of the simple model when events are rare. The approximate crossover point on both bias and coverage appears to be between 0.1 and 0.3. Increasing the rate of outcome 2 has less impact on the bias and coverage of outcome 1 (eg, rate of outcome 2 in the bias for outcome 1 presented in Table 6) than increasing the rate of outcome 1 itself, and vice versa. However, this impact of increasing the rate of the other outcome increases with the rate of the outcome being studied. Spot checks on BGR statistics and history plots for runs of the simulation suggested good convergence of both models for all event rates.

**Table 6:** Simulation study bias results on outcome 1

Average Event Rate Outcome 1	Average Event Rate Outcome 2	0.05	0.1	0.2	0.3	0.4
0.05	Simple	0.0058	0.0057	0.0061	0.0060	0.0062
	Complex	0.0073	0.0067	0.0066	0.0064	0.0064
0.1	Simple	0.0040	0.0042	0.0043	0.0046	0.0047
	Complex	0.0045	0.0045	0.0043	0.0045	0.0043
0.2	Simple	0.0031	0.0032	0.0036	0.0038	0.0039
	Complex	0.0031	0.0031	0.0030	0.0030	0.0030
0.3	Simple	0.0031	0.0033	0.0037	0.0041	0.0043
	Complex	0.0026	0.0026	0.0025	0.0025	0.0025
0.4	Simple	0.0037	0.0041	0.0046	0.0051	0.0056
	Complex	0.0022	0.0023	0.0022	0.0022	0.0022

*Notes.* Greater values indicate worse performance. Simple is simple odds ratio NMA while complex is shared parameter NMA.

**Table 7:** Simulation study bias results on outcome 2

Average Event Rate Outcome 1	Average Event Rate Outcome 2	0.05	0.1	0.2	0.3	0.4
0.05	Simple	0.0061	0.0042	0.0031	0.0029	0.0029
	Complex	0.0079	0.0047	0.0030	0.0025	0.0021
0.1	Simple	0.0061	0.0044	0.0033	0.0030	0.0034
	Complex	0.0076	0.0046	0.0031	0.0024	0.0022
0.2	Simple	0.0063	0.0045	0.0036	0.0035	0.0038
	Complex	0.0076	0.0047	0.0031	0.0025	0.0021
0.3	Simple	0.0064	0.0047	0.0038	0.0039	0.0042
	Complex	0.0074	0.0047	0.0031	0.0025	0.0021
0.4	Simple	0.0065	0.0048	0.0039	0.0040	0.0045
	Complex	0.0074	0.0047	0.0032	0.0025	0.0021

Notes. Greater values indicate worse performance. Simple is simple odds ratio NMA while complex is shared parameter NMA.

**Table 8:** Simulation study mean coverage probability results on outcome 1

Average Event Rate Outcome 1	Average Event Rate Outcome 2	0.05	0.1	0.2	0.3	0.4
0.05	Simple	0.952	0.958	0.940	0.945	0.932
	Complex	0.851	0.874	0.870	0.868	0.856
0.1	Simple	0.956	0.948	0.947	0.928	0.925
	Complex	0.924	0.924	0.927	0.913	0.924
0.2	Simple	0.958	0.947	0.919	0.907	0.888
	Complex	0.945	0.950	0.948	0.948	0.947
0.3	Simple	0.924	0.900	0.853	0.826	0.790
	Complex	0.948	0.942	0.951	0.955	0.952
0.4	Simple	0.844	0.796	0.724	0.663	0.589
	Complex	0.944	0.951	0.952	0.952	0.957

Notes. Greater values indicate better performance. Simple is simple odds ratio NMA while complex is shared parameter NMA.

**Table 9:** Simulation study mean coverage probability results on outcome 2

Average Event Rate Outcome 1	Average Event Rate Outcome 2	0.05	0.1	0.2	0.3	0.4
0.05	Simple	0.945	0.954	0.962	0.949	0.926
	Complex	0.851	0.926	0.961	0.964	0.967
0.1	Simple	0.954	0.951	0.950	0.930	0.888
	Complex	0.867	0.938	0.953	0.959	0.957
0.2	Simple	0.944	0.939	0.925	0.896	0.831
	Complex	0.854	0.928	0.950	0.961	0.970
0.3	Simple	0.932	0.933	0.901	0.852	0.786
	Complex	0.862	0.919	0.953	0.956	0.970
0.4	Simple	0.937	0.927	0.892	0.846	0.744
	Complex	0.847	0.912	0.942	0.952	0.964

Notes. Greater values indicate better performance. Simple is simple odds ratio NMA while complex is shared parameter NMA.

Our simulation results comparing the simple and shared parameter models, when vaguer priors are used in the latter, are presented in Table 10. These were explored for only the cases where data are weakest, namely, the cases where event rates were the rare 0.05 and 0.1. We see that this does not improve either the bias or coverage of the shared parameter models for rare events.

**Table 10:** Vague priors for complex model simulation study results

			Outcome 1 Results		Outcome 2 Results	
	Model	Outcome 2 Average Rate	0.05	0.1	0.05	0.1
		Outcome 1 Average Rate				
Bias	Simple	0.05	0.0060	0.0062	0.0056	0.0040
	Complex		0.0078	0.0077	0.0071	0.0044
	Simple	0.1	0.0042	0.0044	0.0060	0.0043
	Complex		0.0047	0.0047	0.0070	0.0046
Coverage	Simple	0.05	0.959	0.946	0.957	0.958
	Complex		0.860	0.862	0.863	0.932
	Simple	0.1	0.961	0.946	0.947	0.948
	Complex		0.930	0.930	0.863	0.913

Notes. Mean bias and mean coverage for both outcome 1 and outcome 2. Greater coverage and lower bias indicate better performance. Results can be compared with upper left corners of Tables 7 to 10.

## 5 DISCUSSION

In this paper, we have considered the common situation where studies report multiple, potentially competing outcomes and where studies summarize outcomes in different ways. We developed a novel shared parameter model for NMA that accounts for the competing risks and different summaries. We found that this complex model gave almost identical results to a simple model that makes no adjustment. We explored the theoretical reasons why rare events lead to the models giving the same results and used constructed data to identify situations in which the models would disagree, and adjustments would be necessary. We also conducted a simple simulation study to explore the impact of increasing event rates on the bias and coverage of the simple and shared parameter models, confirming that the latter performs better when events are more common and that the crossover point for rates is between 0.1 and 0.3. Although our example was in NMA, the conclusion is general to meta‐analysis of multiple outcomes or where different data summaries are used.

Shared parameter and competing risk NMAs have been previously published but not specifically for the data formats used in our example.^23^, ^38^, ^39^ Woods et al presents a shared parameter model but analyses only a single outcome and combines studies that report mean or median survival with studies that report total numbers of events.^23^ We instead analyze multiple outcomes accounting for competing risks and combine studies that summarize the numbers of events differently. Shared parameter models (such as ours) are specific examples of a more general multiparameter evidence synthesis (MPES) framework.^38^, ^39^ Competing risk NMA has been studied in the literature,^1^ but this earlier work assumes that all studies report data in the same way. Our work can be considered an extension of previous developments in shared parameter models and competing risks within the more general MPES framework. However, there are several issues to consider when drawing conclusions from our study.

Meta‐analysis and NMA are the recommended approach for treatment effect estimation and indirect comparison by health care authorities and international medical decision‐making societies.^7^, ^8^ It is therefore important to ensure that unbiased methods are used. Meta‐analysis and NMA results may also be used as inputs to cost‐effectiveness models.^13^ As treatment effects on multiple outcomes must be simultaneously estimated, it is important to account for competing risks; this is relevant to our atrial fibrillation example as the estimates were used in a cost‐effectiveness model.^26^, ^27^ Also, although clinical conclusions may not be sensitive to adjustment for competing risks or different data summaries, the cost‐effectiveness results may be impacted. This has been the finding in work considering different synthesis models in type 1 diabetes where clinical conclusions were unaffected but cost‐effectiveness conclusions changed.^14^ The correlation matrix between apixaban hazard ratios of events is presented in Table 11, with other matrices in the Supporting Information. This demonstrates that the hazard ratio estimates were correlated. The simple model assumes these correlations to be zero, and an economic model based could be biased by omission of this correlation.

**Table 11:** Correlation matrix for hazard ratios of events of interest for apixaban

	MI	Ischaemic Stroke	Death (All Causes)	Clinically Relevant Bleeding
MI	1.000	0.028	−0.007	0.017
Ischaemic stroke	0.028	1.000	0.012	−0.027
Death (all causes)	−0.007	0.012	1.000	−0.022
Clinically relevant bleeding	0.017	−0.027	−0.022	1.000

Our finding from both the constructed data and simulation study was that event rates above 0.2 might warrant consideration of different data summaries and competing risks. Below this level, however, our simulation study suggests that the simple odds ratio model may be preferred. A possible explanation for the superior performance on rare event data of the simple model is influence of the prior distributions, which have greater impact due to the larger number of parameters in the shared parameter model. However, our simulation study for vague priors in the shared parameter model indicates that bias and coverage are not improved when vaguer priors are used. In addition, convergence can become an issue when vague priors are assumed for the shared parameter model.

The sensitivity to competing risks has been identified in NMA examples,^4^ and our approximate threshold 0.2 is in line with a previous informal assessment to the sensitivity of using odds and hazard ratios.^32^ Although event rates in our atrial fibrillation example were below the 0.2 threshold, rates above this level are common in other fields, including response and remission in depression,^9^ annualized relapse rates in relapsing remitting multiple sclerosis,^11^ and 5‐year survival and mortality rates in a range of cancers.^12^ Despite this sensitivity, high profile NMAs continue not to adjust for competing risks or consider different data summaries when analyzing multiple outcomes.^10^

There are important limitations to our analysis. We did not use theoretical analysis to estimate the event rate threshold below which competing risks and different data summaries can be ignored; our informal threshold came only from constructed data analyses and a simple simulation study. Estimating this threshold, or identifying conditions under which competing risks and data summaries cannot be ignored, by asymptotic arguments may be worthwhile future research. Although our simulation study, unlike the constructed data example, explored differential event rates, we did not formally assess correlation.

## 6 CONCLUSIONS

In this paper, we have considered a NMA where the RCTs report results on competing risks in different formats. We developed a novel shared parameter model accounting for all these complications. Applying this model to a synthesis of interventions for atrial fibrillation however, results were almost identical to a model without the necessary adjustments. We used theoretical arguments to demonstrate that this is due to the rareness of events. We further used constructed data examples and a simulation study to find situations in which the adjustments may become necessary. We recommend analysts to consider the absolute event rates and only use a simple model ignoring data types and competing risks, if all of these underlying events are rare (below our threshold of approximately 0.2). If one or more of the absolute event rates approaches or exceeds our informal threshold, it is necessary to account for data types and competing risks through a shared parameter model in order to avoid biased estimates. A more complete simulation study could simulate different numbers of treatments or outcomes, multiarmed trials, sample sizes, and more varied event rates. This was outside the scope of the present study but could be a useful piece of future research.

## CONFLICT OF INTEREST

The author reported no conflict of interest.

## Supporting information

Table S1 List of all treatments included in the network meta‐analysis
Table S2 Comparison of constructed data results for edoxaban
Table S3 Comparison of constructed data results for rivaroxaban
Table S4 Correlation matrix for hazard ratios of events of interest for dabigatran
Table S5 Correlation matrix for hazard ratios of events of interest for edoxaban
Table S6 Correlation matrix for hazard ratios of events of interest for rivaroxaban
Figure S1 Network diagrams for the four outcomes of interest
Figure S2 Comparison of estimated adjusted HR (dotted) and simple OR (solid) for edoxaban under constructed data scenarios. Thin lines represent upper and lower 95% credible interval limits.
Figure S3 Comparison of estimated adjusted HR (dotted) and simple OR (solid) for rivaroxaban under constructed data scenarios. Thin lines represent upper and lower 95% credible interval limits.

## Data Availability

The data to produce the base case results of the DOAC example, along with necessary OpenBUGS code and initial values, are provided in the Supporting Information. We have also provided OpenBUGS code, two sets of initial values, and data from one of the simulated datasets so that interested readers can adapt our analysis to their examples. The simulated dataset is smaller and more efficient for adaptation.
