# Supplementary material for: Shared parameter model for competing risks and different data summaries in meta‐analysis: Implications for common and rare outcomes
Source: Res Synth Methods. 2019 Aug 22;11(1):91–104. doi: 10.1002/jrsm.1371 (PMC7003901; doi:10.1002/jrsm.1371)
Supplement: Supplementary file 1 — Table S1 List of all treatments included in the network meta‐analysis Table S2 Comparison of constructed data results for edoxaban Table S3 Comparison of constructed data results for rivaroxaban Table S4 Correlation matrix for hazard ratios of events of interest for dabigatran Table S5 Correlation matrix for hazard ratios of events of interest for edoxaban Table S6 Correlation matrix for hazard ratios of events of interest for rivaroxaban Figure S1 Network diagrams for the four outcomes of interest Figure S2 Comparison of estimated adjusted HR (dotted) and simple OR (solid) for edoxaban under constructed data scenarios. Thin lines represent upper and lower 95% credible interval limits. Figure S3 Comparison of estimated adjusted HR (dotted) and simple OR (solid) for rivaroxaban under constructed data scenarios. Thin lines represent upper and lower 95% credible interval limits. [file JRSM-11-91-s001.docx]

## Appendix 1. Additional details of the network meta-analysis

Table 1 lists the 25 treatments that were included in the network meta-analysis. Note that these networks are based on 20 RCTs identified in a systematic review; 3 RCTs using subclinical INR ranges for warfarin in elderly patients were excluded. Network diagrams for the four outcomes of interest are presented in Figure 1.

Note that our outcome of clinically relevant bleeding was a combination of “major bleeding” and “clinically relevant non-major bleeding”, depending on what was reported. Also note that not all RCTs reported all 17 outcomes, and indeed the outcome definitions are overlapping (e.g. cardiovascular death and all-cause death), and we will not model the correlation between outcomes with similar definitions explicitly.

Table 1 List of all treatments included in the network meta-analysis*

| 1 | Coumarin (INR 2-3) |
| --- | --- |
| 2 | Antiplatelet (>=150mg od) |
| 3 | Apixaban (2.5mg bd) |
| 4 | **Apixaban (5mg bd)** |
| 5 | Betrixaban (40mg od) |
| 6 | Betrixaban (60mg od) |
| 7 | Betrixaban (80mg od) |
| 8 | Antiplatelet (<150mg od) |
| 9 | Coumarin (INR 3-4) |
| 10 | Dabigatran (110mg bd) |
| 11 | **Dabigatran (150mg bd)** |
| 12 | Dabigatran (150mg bd) + Aspirin (325mg bd) |
| 13 | Dabigatran (150mg bd) + Aspirin (81mg bd) |
| 14 | Dabigatran (300mg bd) |
| 15 | Dabigatran (300mg bd) + Aspirin (325mg bd) |
| 16 | Dabigatran (300mg bd) + Aspirin (81mg bd) |
| 17 | Dabigatran (50mg bd) |
| 18 | Dabigatran (50mg bd) + Aspirin (325mg bd) |
| 19 | Dabigatran (50mg bd) + Aspirin (81mg bd) |
| 20 | Edoxaban (30mg bd) |
| 21 | Edoxaban (30mg od) |
| 22 | Edoxaban (45mg od) |
| 23 | Edoxaban (60mg bd) |
| 24 | **Edoxaban (60mg od)** |
| 25 | **Rivaroxaban (20mg od)** |

*Treatments of interest for our analysis are written in bold typeface.

Figure 1 Network diagrams for the four outcomes of interest

| **Ischaemic stroke** |
| --- |
| **** |
| **Myocardial infarction** |
| **** |

| **All-cause mortality** |
| --- |
| **** |
| **Clinically relevant bleeding** |
| **** |

## Appendix 2. Equivalence of competing and non-competing risks NMA under rare events

We consider the conditions under which the probability of an event in a competing risks NMA, as described in section 3.2, becomes the same as the probability under non-competing risks NMA. Suppressing the subscripts for arm $k$ and study $i$, the probability of event m before time D under competing risks is

$$P_{m}^{C}\left( D \right)=\int_{0}^{D} \lambda_{m}e^{-t\sum_{l=1}^{M} \lambda_{l}}dt$$

$$=\frac{\lambda_{m}}{\sum\lambda_{l}}-\frac{\lambda_{m}e^{-D\sum\lambda_{l}}}{\sum\lambda_{l}}$$

Under non-competing risks this is

$$P_{m}\left( D \right)=1-e^{-\lambda_{m}D}$$

These probabilities become the same if the hazard of other events ($l\neq m$) tend to zero as, under this rare events assumption, we get $\sum\lambda_{l}\to\lambda_{m}$ and $P_{m}^{C}\left( D \right)\to P_{m}\left( D \right)$. This makes intuitive sense as other events will not interfere with the event $m$.

It is also intuitive that $\lambda_{m}\to0$ would not be sufficient to remove the effect of competing risks if the other $\lambda_{l}$ did not tend to 0. This is proved by first noting

$$\lim_{\lambda_{m}\to0} P_{m}(D)=0$$

And then calculating

$$\lim_{\lambda_{m}\to0} P_{m}^{C}(D)=0-\frac{\lambda_{m}e^{-D(\lambda_{m}+\sum_{l\neq m} \lambda_{l})}}{\lambda_{m}+\sum_{l\neq m} \lambda_{l}}$$

Taking the derivative with respect to $\lambda_{m}$ in the numerator and denominator gives

$$-\frac{e^{-D(\lambda_{m}+\sum_{l\neq m} \lambda_{l})}-D\lambda_{m}e^{-D(\lambda_{m}+\sum_{l\neq m} \lambda_{l})}}{1}$$

Taking the limit as $\lambda_{m}\to0$ gives

$$\lim_{\lambda_{m}\to0} P_{m}^{C}(D)=-e^{-D\sum_{l\neq m} \lambda_{l}}$$

Which is not the same as $\lim_{\lambda_{m}\to0} P_{m}(D)$. This confirms the intuition that $\lambda_{m}\to0$ for our event of interest is not sufficient to remove the effects of competing risks; it is the other events that must be rare. Note that these findings can be generalised to non-constant hazards models $\lambda_{m}=\lambda_{m}(t)$ by putting an upper bound on the timewise integrals and allowing the bound to tend to zero as events become increasingly rare.

## Appendix 3. Model comparison for edoxaban and rivaroxaban

Table 2 Comparison of constructed data results for edoxaban

| **Scenario** | **Outcome** | **Adjusted HR****  **Mean (95% CrI)** | **Simple OR*****  **Mean (95% CrI)** | **Event rate****** |
| --- | --- | --- | --- | --- |
| **Base rate** | Ischemic stroke | 0.998 (0.829, 1.2) | 1 (0.837, 1.21) | 0.0125 |
|  | MI | 0.938 (0.745, 1.18) | 0.958 (0.757, 1.21) | 0.00715 |
|  | Death (all causes) | 0.914 (0.827, 1.01) | 0.911 (0.822, 1.01) | 0.0409 |
|  | Clinically relevant bleeding | 0.881 (0.825, 0.944) | 0.848 (0.785, 0.916) | 0.0843 |
| **Inflate by 2** | Ischemic stroke | 1 (0.879, 1.13) | 1 (0.879, 1.14) | 0.025 |
|  | MI | 0.951 (0.8, 1.13) | 0.957 (0.806, 1.13) | 0.0143 |
|  | Death (all causes) | 0.909 (0.849, 0.977) | 0.9 (0.831, 0.973) | 0.0819 |
|  | Clinically relevant bleeding | 0.882 (0.84, 0.925) | 0.787 (0.738, 0.84) | 0.169 |
| **Inflate by 5** | Ischemic stroke | 1 (0.925, 1.09) | 1.01 (0.922, 1.1) | 0.0625 |
|  | MI | 0.953 (0.857, 1.06) | 0.953 (0.853, 1.06) | 0.0357 |
|  | Death (all causes) | 0.879 (0.839, 0.921) | 0.825 (0.771, 0.883) | 0.205 |
|  | Clinically relevant bleeding | 1 (0.97, 1.04) | 1.04 (0.769, 1.41) | 0.378 |
| **Inflate by 10** | Ischemic stroke | 1.01 (0.948, 1.06) | 1.01 (0.939, 1.08) | 0.125 |
|  | MI | 0.953 (0.884, 1.02) | 0.948 (0.872, 1.03) | 0.0715 |
|  | Death (all causes) | 1.83 (0.263, 20.3) | 91.2 (9.88e-05, 1.39e+09) | 0.372 |
|  | Clinically relevant bleeding | 1.01 (0.972, 1.04) | 1.47 (1.08, 2) | 0.384 |
| **Inflate by 20** | Ischemic stroke | 1.01 (0.965, 1.05) | 1.01 (0.946, 1.09) | 0.25 |
|  | MI | 0.944 (0.894, 0.993) | 0.932 (0.871, 0.998) | 0.143 |
|  | Death (all causes) | 1.89 (0.272, 20.2) ***** | 83.2 (9.32e-05, 1.16e+09) ***** | 0.372 |
|  | Clinically relevant bleeding | 1.01 (0.979, 1.04) | 2.7 (1.84, 4) | 0.393 |

* Results are shaded if the 95% credible intervals do not overlap

** Adjusted HR is the hazard ratio estimated by the model accounting for competing risks and differently reported data.

*** Simple OR is the odds ratio estimated by the model that disregards competing risks and differently reported data.

**** Averaged over all arms and all trials.

*****Anomalous results due to high event rate.

Table 3 Comparison of constructed data results for rivaroxaban

| **Scenario** | **Outcome** | **Adjusted HR****  **Mean (95% CrI)** | **Simple OR*****  **Mean (95% CrI)** | **Event rate****** |
| --- | --- | --- | --- | --- |
| **Base rate** | Ischemic stroke | 0.916 (0.734, 1.15) | 0.926 (0.74, 1.16) | 0.0108 |
|  | MI | 0.791 (0.608, 1.04) | 0.8 (0.613, 1.04) | 0.00731 |
|  | Death (all causes) | 0.824 (0.686, 0.985) | 0.83 (0.689, 1) | 0.0151 |
|  | Clinically relevant bleeding | 1.05 (0.976, 1.13) | 1.07 (0.985, 1.16) | 0.11 |
| **Inflate by 2** | Ischemic stroke | 0.921 (0.787, 1.08) | 0.923 (0.785, 1.09) | 0.0216 |
|  | MI | 0.793 (0.66, 0.951) | 0.797 (0.661, 0.962) | 0.0146 |
|  | Death (all causes) | 0.829 (0.726, 0.945) | 0.825 (0.72, 0.941) | 0.0301 |
|  | Clinically relevant bleeding | 1.05 (0.997, 1.1) | 1.09 (1.02, 1.17) | 0.221 |
| **Inflate by 5** | Ischemic stroke | 0.921 (0.833, 1.02) | 0.92 (0.828, 1.02) | 0.0539 |
|  | MI | 0.794 (0.708, 0.893) | 0.79 (0.7, 0.893) | 0.0366 |
|  | Death (all causes) | 0.82 (0.756, 0.891) | 0.806 (0.736, 0.882) | 0.0753 |
|  | Clinically relevant bleeding | 0.997 (0.965, 1.03) | 138 (0.00024, 1.78e+09) | 0.511 |
| **Inflate by 10** | Ischemic stroke | 0.918 (0.856, 0.982) | 0.908 (0.839, 0.984) | 0.108 |
|  | MI | 0.787 (0.725, 0.855) | 0.771 (0.705, 0.843) | 0.0731 |
|  | Death (all causes) | 0.802 (0.756, 0.85) | 0.765 (0.712, 0.821) | 0.151 |
|  | Clinically relevant bleeding | 0.997 (0.964, 1.03) | 131 (0.000221, 1.82e+09) ***** | 0.511 |
| **Inflate by 20** | Ischemic stroke | 0.904 (0.86, 0.949) | 0.875 (0.819, 0.935) | 0.216 |
|  | MI | 0.767 (0.724, 0.813) | 0.725 (0.676, 0.779) | 0.146 |
|  | Death (all causes) | 0.727 (0.696, 0.759) | 0.597 (0.557, 0.641) | 0.301 |
|  | Clinically relevant bleeding | 0.997 (0.965, 1.03) | 124 (0.000179, 1.49e+09) | 0.511 |

* Results are shaded if the 95% credible intervals do not overlap

** Adjusted HR is the hazard ratio estimated by the model accounting for competing risks and differently reported data.

*** Simple OR is the odds ratio estimated by the model that disregards competing risks and differently reported data.

**** Averaged over all arms and all trials.

*****Anomalous results due to high event rate.

Figure 2 Comparison of estimated adjusted HR (dotted) and simple OR (solid) for edoxaban under constructed data scenarios. Thin lines represent upper and lower 95% credible interval limits.


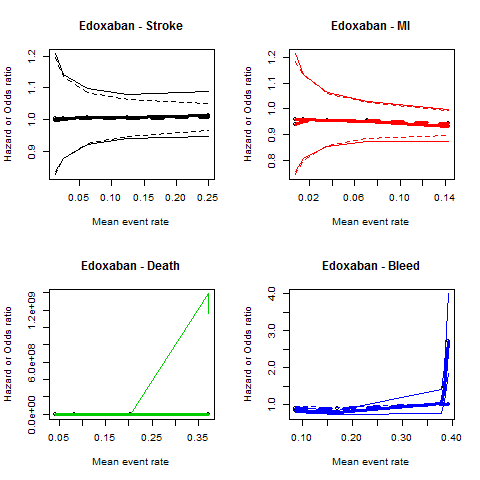


Figure 3 Comparison of estimated adjusted HR (dotted) and simple OR (solid) for rivaroxaban under constructed data scenarios. Thin lines represent upper and lower 95% credible interval limits.


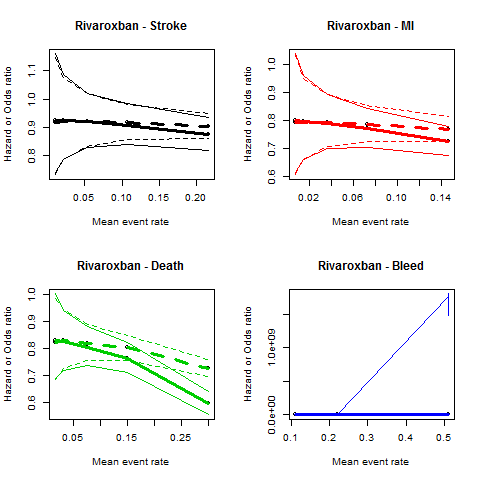


## Appendix 4. Correlation matrices for hazard ratios for dabigatran, edoxaban, and rivaroxaban

Table 4 Correlation matrix for hazard ratios of events of interest for dabigatran

|  | **MI** | **Ischemic stroke** | **Death (all causes)** | **Clinically relevant bleeding** |
| --- | --- | --- | --- | --- |
| **MI** | 1.000 | -0.003 | 0.019 | 0.020 |
| **Ischemic stroke** | -0.003 | 1.000 | 0.006 | -0.005 |
| **Death (all causes)** | 0.019 | 0.006 | 1.000 | 0.025 |
| **Clinically relevant bleeding** | 0.020 | -0.005 | 0.025 | 1.000 |

Table 5 Correlation matrix for hazard ratios of events of interest for edoxaban

|  | **MI** | **Ischemic stroke** | **Death (all causes)** | **Clinically relevant bleeding** |
| --- | --- | --- | --- | --- |
| **MI** | 1.000 | -0.003 | -0.004 | 0.019 |
| **Ischemic stroke** | -0.003 | 1.000 | -0.009 | -0.010 |
| **Death (all causes)** | -0.004 | -0.009 | 1.000 | 0.001 |
| **Clinically relevant bleeding** | 0.019 | -0.010 | 0.001 | 1.000 |

Table 6 Correlation matrix for hazard ratios of events of interest for rivaroxaban

|  | **MI** | **Ischemic stroke** | **Death (all causes)** | **Clinically relevant bleeding** |
| --- | --- | --- | --- | --- |
| **MI** | 1.000 | 0.007 | -0.020 | -0.002 |
| **Ischemic stroke** | 0.007 | 1.000 | -0.018 | -0.001 |
| **Death (all causes)** | -0.020 | -0.018 | 1.000 | 0.019 |
| **Clinically relevant bleeding** | -0.002 | -0.001 | 0.019 | 1.000 |

## Appendix 5. BUGS code and simulated data for the shared parameter model adjusting for competing risks and different data summaries.

We provide below the OpenBUGS script for the shared parameter model. Data and initial values are provided for one of the simulated datasets where there were 3 treatments, 10 trials (4 of type 1, 3 of type 2, and 3 of type 3.), and 2 outcomes, both of which had event rates of 0.05.

*## Data ################################################################*

*list(ns=1.00000E+01, ns.type1=4.00000E+00, ns.type2=3.00000E+00, ns.type3=3.00000E+00, type1=c(1.00000E+00, 2.00000E+00, 3.00000E+00, 4.00000E+00), type2=c(5.00000E+00, 6.00000E+00, 7.00000E+00), type3=c(8.00000E+00, 9.00000E+00, 1.00000E+01), study1.j=1.00000E+00, study2.j=2.00000E+00, study3.j=3.00000E+00, study4.j=4.00000E+00, study1.R=c(4.00000E+00, 1.00000E+01), study2.R=c(8.00000E+00, 9.00000E+00), study3.R=c(2.00000E+00, 1.80000E+01), study4.R=c(1.20000E+01, 1.20000E+01), n= structure(.Data= c(1.00000E+02, 1.00000E+02, 1.00000E+02, 1.00000E+02, 1.00000E+02, 1.00000E+02, 1.00000E+02, 1.00000E+02, 1.00000E+02, 1.00000E+02, 1.00000E+02, 1.00000E+02, 1.00000E+02, 1.00000E+02, 1.00000E+02, 1.00000E+02, 1.00000E+02, 1.00000E+02, 1.00000E+02, 1.00000E+02), .Dim=c(10, 2)), E= structure(.Data= c(1.00000E+02, 1.00000E+02, 1.00000E+02, 1.00000E+02, 1.00000E+02, 1.00000E+02, 1.00000E+02, 1.00000E+02, 1.00000E+02, 1.00000E+02, 1.00000E+02, 1.00000E+02, 1.00000E+02, 1.00000E+02, 1.00000E+02, 1.00000E+02, 1.00000E+02, 1.00000E+02, 1.00000E+02, 1.00000E+02), .Dim=c(10, 2)), f= structure(.Data= c(1.00000E+00, 1.00000E+00, 1.00000E+00, 1.00000E+00, 1.00000E+00, 1.00000E+00, 1.00000E+00, 1.00000E+00, 1.00000E+00, 1.00000E+00, 1.00000E+00, 1.00000E+00, 1.00000E+00, 1.00000E+00, 1.00000E+00, 1.00000E+00, 1.00000E+00, 1.00000E+00, 1.00000E+00, 1.00000E+00), .Dim=c(10, 2)), na=c(2.00000E+00, 2.00000E+00, 2.00000E+00, 2.00000E+00, 2.00000E+00, 2.00000E+00, 2.00000E+00, 2.00000E+00, 2.00000E+00, 2.00000E+00), nt=3.00000E+00, t= structure(.Data= c(1.00000E+00, 2.00000E+00, 1.00000E+00, 3.00000E+00, 1.00000E+00, 2.00000E+00, 2.00000E+00, 3.00000E+00, 1.00000E+00, 2.00000E+00, 1.00000E+00, 3.00000E+00, 1.00000E+00, 3.00000E+00, 2.00000E+00, 3.00000E+00, 1.00000E+00, 3.00000E+00, 1.00000E+00, 2.00000E+00), .Dim=c(10, 2)), n.study1.outcomes=2.00000E+00, n.study2.outcomes=2.00000E+00, n.study3.outcomes=2.00000E+00, n.study4.outcomes=2.00000E+00, study1.outcomes=c(1.00000E+00, 2.00000E+00), study2.outcomes=c(1.00000E+00, 2.00000E+00), study3.outcomes=c(1.00000E+00, 2.00000E+00), study4.outcomes=c(1.00000E+00, 2.00000E+00), r= structure(.Data= c(1.00000E+00, 3.00000E+00, 1.00000E+00, 3.00000E+00, 6.00000E+00, 2.00000E+00, 6.00000E+00, 2.00000E+00, 2.00000E+00, 0.00000E+00, 2.00000E+00, 0.00000E+00, 5.00000E+00, 7.00000E+00, 3.00000E+00, 9.00000E+00, 2.00000E+00, 6.00000E+00, 1.00000E+01, 5.00000E+00, 4.00000E+00, 4.00000E+00, 4.00000E+00, 7.00000E+00, 8.00000E+00, 1.00000E+00, 2.00000E+00, 4.00000E+00, 6.00000E+00, 5.00000E+00, 6.00000E+00, 1.10000E+01, 5.00000E+00, 4.00000E+00, 5.00000E+00, 1.00000E+01, 9.00000E+00, 4.00000E+00, 8.00000E+00, 7.00000E+00), .Dim=c(10, 2, 2)), m.outcomes=2.00000E+00, m=2.00000E+00, outcomes.to.save=c(1.00000E+00, 2.00000E+00), n.to.save=1.00000E+00)*

*## Initial values for two chains #################################################*

*list(mu= structure(.Data= c(1.00000E-01, 1.00000E-01, 1.00000E-01, 1.00000E-01, 1.00000E-01, 1.00000E-01, 1.00000E-01, 1.00000E-01, 1.00000E-01, 1.00000E-01, 1.00000E-01, 1.00000E-01, 1.00000E-01, 1.00000E-01, 1.00000E-01, 1.00000E-01, 1.00000E-01, 1.00000E-01, 1.00000E-01, 1.00000E-01), .Dim=c(10, 2)), d= structure(.Data= c( NA, NA, 1.00000E-01, 1.00000E-01, 1.00000E-01, 1.00000E-01), .Dim=c(3, 2)))*

*list(mu= structure(.Data= c(5.00000E-01, 5.00000E-01, 5.00000E-01, 5.00000E-01, 5.00000E-01, 5.00000E-01, 5.00000E-01, 5.00000E-01, 5.00000E-01, 5.00000E-01, 5.00000E-01, 5.00000E-01, 5.00000E-01, 5.00000E-01, 5.00000E-01, 5.00000E-01, 5.00000E-01, 5.00000E-01, 5.00000E-01, 5.00000E-01), .Dim=c(10, 2)), d= structure(.Data= c( NA, NA, 1.00000E-01, 1.00000E-01, 1.00000E-01, 1.00000E-01), .Dim=c(3, 2)))*

*## OpenBUGS code ############################################################*

*# Model to estimate log hazard ratios for effect of treatment on multiple outcomes*

*# Accounts for competing risks, differences in study duration, and the three*

*# types of outcome data*

*# i is the outcome and there are m of these*

*# j is study and there are ns of these studies and m outcomes*

*# nt is the number of treatments*

*# ns is the number of studies*

*# type1[] is a vector containing a list of studies that are of type 1 eg. c(1,2,3,4)*

*# ns.type1 is the number of studies of type 1 (the length of the type1[] vector)*

*# r[j,k,i] are the number of events of type i in arm k of study j*

*# E[j,k] contains the exposure for study j, arm k*

*# na[j] are the number of arms in study j*

*# t[j,k] is the treatment in arm k of study j*

*# studyx.R[j,k] is total number of events (of any type) i STUDYX of type 1.*

*# n[j,k] are the number of patients in arm k of study j*

*# type2[] is a vector containing a list of studies that are of type 2 eg. c(2,3,4,9,13)*

*# f[j,k] contains mean follow-up time, if available, for study j and arm k and median otherwise*

*# mu[j,i] is the baseline for study j for outcome type i*

*# d[t[j,k],i] is the log hazard ratio relative to treatment 1 for the treatment t[j,k] and outcome i*

*# Includes four separate cstudies that report on 1st event data (type1)*

*# These are STUDY1, STUDY2, STUDY3, STUDY4*

*# Additional studies can be added by duplicating the code and labelling, for example, STUDY5*

*# Outcome indicators are in study1.outcomes, study2.outcomes, etc.*

*# Number of outcomes are n.study1.outcomes, n.study2.outcomes, etc.*

*# Study indicators are study1.j, study2.j, etc.*

*# Total number of events in each arm and of each event in particular are in study1.R and study2.r, etc.*

*model{*

*for(j in 1:ns){*

*for(k in 1:na[j]){*

*# Set equal to obsereved E*

*E.completed[j,k] <- E[j,k]*

*}*

*}*

*# Loop over all outcomes and studies*

*for(i in 1:m){*

*for(j in 1:ns){*

*mu[j,i] ~ dnorm(0, 0.5) # vague priors for trial baselines*

*# Model for the log hazards and log hazard ratios*

*for(k in 1:na[j]){*

*log(lambda[j,k,i]) <- mu[j,i] + d[t[j,k],i] - d[t[j,1],i]*

*}*

*}*

*d[1,i] <- 0 # treatment effect is zero for reference treatment*

*for(k in 2:nt){ d[k,i] ~ dnorm(0, 0.5) } # vague priors for treatment effects*

*}*

*# Type 1 (competing risks) data*

*# STUDY1*

*for(k in 1:na[study1.j]){*

*# Trial specific set of lambdas*

*for(i in 1:n.study1.outcomes){*

*study1.lambda[k,i]<-lambda[study1.j,k,study1.outcomes[i]]*

*}*

*sum.lambda[study1.j,k]<-sum(study1.lambda[k,])*

*# Divide the lambdas by their sum to scale them for the multinomial likelihood*

*for(i in 1:n.study1.outcomes){*

*study1.lambda.scaled[k,i]<-study1.lambda[k,i]/sum.lambda[study1.j,k]*

*}*

*study1.R[k] ~ dpois(theta1[study1.j,k])*

*theta1[study1.j,k]<-E.completed[study1.j,k]*sum.lambda[study1.j,k]*

*# Multinomial likelihood*

*study1.r[k,1:n.study1.outcomes]~dmulti(study1.lambda.scaled[k,1:n.study1.outcomes],study1.R[k])*

*}*

*# STUDY2*

*for(k in 1:na[study2.j]){*

*# Trial specific set of lambdas*

*for(i in 1:n.study2.outcomes){*

*study2.lambda[k,i]<-lambda[study2.j,k,study2.outcomes[i]]*

*}*

*sum.lambda[study2.j,k]<-sum(study2.lambda[k,])*

*# Divide the lambdas by their sum to scale them for the multinomial likelihood*

*for(i in 1:n.study2.outcomes){*

*study2.lambda.scaled[k,i]<-study2.lambda[k,i]/sum.lambda[study2.j,k]*

*}*

*study2.R[k] ~ dpois(theta1[study2.j,k])*

*theta1[study2.j,k]<-E.completed[study2.j,k]*sum.lambda[study2.j,k]*

*# Multinomial likelihood*

*study2.r[k,1:n.study2.outcomes]~dmulti(study2.lambda.scaled[k,1:n.study2.outcomes],study2.R[k])*

*}*

*# STUDY3*

*for(k in 1:na[study3.j]){*

*# Trial specific set of lambdas*

*for(i in 1:n.study3.outcomes){*

*study3.lambda[k,i]<-lambda[study3.j,k,study3.outcomes[i]]*

*}*

*sum.lambda[study3.j,k]<-sum(study3.lambda[k,])*

*# Divide the lambdas by their sum to scale them for the multinomial likelihood*

*for(i in 1:n.study3.outcomes){*

*study3.lambda.scaled[k,i]<-study3.lambda[k,i]/sum.lambda[study3.j,k]*

*}*

*study3.R[k] ~ dpois(theta1[study3.j,k])*

*theta1[study3.j,k]<-E.completed[study3.j,k]*sum.lambda[study3.j,k]*

*# Multinomial likelihood*

*study3.r[k,1:n.study3.outcomes]~dmulti(study3.lambda.scaled[k,1:n.study3.outcomes],study3.R[k])*

*}*

*# STUDY4*

*for(k in 1:na[study4.j]){*

*# Trial specific set of lambdas*

*for(i in 1:n.study4.outcomes){*

*study4.lambda[k,i]<-lambda[study4.j,k,study4.outcomes[i]]*

*}*

*sum.lambda[study4.j,k]<-sum(study4.lambda[k,])*

*# Divide the lambdas by their sum to scale them for the multinomial likelihood*

*for(i in 1:n.study4.outcomes){*

*study4.lambda.scaled[k,i]<-study4.lambda[k,i]/sum.lambda[study4.j,k]*

*}*

*study4.R[k] ~ dpois(theta1[study4.j,k])*

*theta1[study4.j,k]<-E.completed[study4.j,k]*sum.lambda[study4.j,k]*

*# Multinomial likelihood*

*study4.r[k,1:n.study4.outcomes]~dmulti(study4.lambda.scaled[k,1:n.study4.outcomes],study4.R[k])*

*}*

*for(j in 1:ns.type2) {*

*for(k in 1:na[type2[j]]){*

*# Impute the missing mean/median*

*f.completed[type2[j],k] <- f[type2[j],k]*

*r[type2[j],k,m] ~ dpois(theta.m[type2[j],k]) # Separate mortality likelihood*

*theta.m[type2[j],k]<- E.completed[type2[j],k]*lambda[type2[j],k,m]*

*for(i in 1:(m-1)){*

*r[type2[j],k,i] ~ dbin(p[type2[j],k,i],n[type2[j],k]) # Independent non-mortality events*

*cloglog(p[type2[j],k,i]) <- log(f.completed[type2[j],k]) + log(lambda[type2[j],k,i])*

*}*

*}*

*}*

*for(j in 1:ns.type3){*

*for(i in 1:m){*

*for(k in 1:na[type3[j]]){*

*r[type3[j],k,i] ~ dpois(theta3[type3[j],k,i])*

*theta3[type3[j],k,i] <- E.completed[type3[j],k] * lambda[type3[j],k,i]*

*}*

*}*

*}*

*# pairwise HRs and LHRs for all possible pair-wise comparisons*

*for(i in 1:m){*

*for (c in 1:(nt-1)) {*

*for (k in (c+1):nt) {*

*hr[c,k,i] <- exp(d[k,i] - d[c,i])*

*lhr[c,k,i] <- (d[k,i]-d[c,i])*

*}*

*}*

*}*

*# Only report outcomes of interest*

*for(i in 1:n.to.save)*

*{*

*for(k in 1:nt)*

*{*

*d.save[k,i]<-d[k,outcomes.to.save[i]]*

*}*

*}*

*}*

## Appendix 6. BUGS code and DOACs data for the shared parameter model adjusting for competing risks and different data summaries.

We provide below the OpenBUGS script for the shared parameter model along with initial values and data for the DOACs data. This is a specific example of the code given in Appendix 5 and allows the reader to reproduce the base case (no inflation of event rates) results. The data in this case is larger and messier so for learning purposes (and adaptation) we recommend using the stylised example from Appendix 5.

*## Data ################################################################*

*list(nt=2.50000E+01, ns=2.00000E+01, type2=c(2.00000E+00, 4.00000E+00, 5.00000E+00, 6.00000E+00, 7.00000E+00, 8.00000E+00, 1.00000E+01, 1.30000E+01, 1.40000E+01, 1.60000E+01, 1.70000E+01, 1.80000E+01, 2.00000E+01), type3=c(9.00000E+00, 1.90000E+01), ns.type2=1.30000E+01, ns.type3=2.00000E+00, r= structure(.Data= c(4.20000E+01, 5.55000E+02, 4.81000E+02, 1.10000E+01, 2.30000E+01, 1.58000E+02, NA, NA, NA, NA, NA, NA, NA, NA, NA, 1.50000E+01, 9.30000E+01, 9.00000E+01, 6.44000E+02, 5.68000E+02, 7.00000E+00, 3.60000E+01, 1.59000E+02, NA, NA, NA, NA, NA, NA, NA, NA, NA, 5.00000E+00, 1.01000E+02, NA, NA, NA, NA, NA, NA, NA, NA, NA, NA, NA, NA, NA, NA, NA, NA, NA, NA, NA, NA, NA, NA, NA, NA, NA, NA, NA, NA, NA, NA, NA, NA, NA, NA, NA, NA, NA, NA, NA, NA, NA, NA, NA, NA, NA, NA, NA, NA, NA, NA, NA, NA, NA, NA, NA, NA, NA, NA, NA, NA, NA, NA, NA, NA, NA, NA, NA, NA, NA, NA, NA, NA, NA, NA, NA, NA, NA, NA, NA, NA, NA, NA, NA, NA, NA, NA, NA, NA, NA, NA, NA, NA, NA, NA, NA, NA, NA, NA, NA, NA, NA, NA, NA, NA, NA, NA, NA, NA, NA, NA, NA, NA, NA, NA, NA, NA, NA, NA, NA, NA, NA, NA, NA, NA, NA, NA, NA, NA, NA, NA, NA, NA, NA, NA, NA, NA, NA, 2.00000E+00, NA, NA, NA, 1.20000E+01, 2.00000E+00, 3.00000E+00, NA, NA, NA, NA, NA, NA, NA, NA, NA, NA, 2.10000E+01, NA, NA, NA, 3.00000E+00, 0.00000E+00, 1.00000E+00, NA, NA, NA, NA, NA, NA, NA, NA, NA, NA, NA, NA, NA, NA, NA, NA, NA, NA, NA, NA, NA, NA, NA, NA, NA, NA, NA, NA, NA, NA, NA, NA, NA, NA, NA, NA, NA, NA, NA, NA, NA, NA, NA, NA, NA, NA, NA, NA, NA, NA, NA, NA, NA, NA, NA, NA, NA, NA, NA, NA, NA, NA, NA, NA, NA, NA, NA, NA, NA, NA, NA, NA, NA, NA, NA, NA, NA, NA, NA, NA, NA, NA, NA, NA, NA, NA, NA, NA, NA, NA, NA, NA, NA, NA, NA, NA, NA, NA, NA, NA, NA, NA, NA, NA, NA, NA, NA, NA, NA, NA, NA, NA, NA, NA, NA, NA, NA, NA, NA, NA, NA, NA, NA, NA, NA, NA, NA, NA, NA, NA, NA, NA, NA, NA, NA, NA, NA, NA, NA, NA, NA, NA, NA, NA, NA, 3.00000E+00, NA, 4.20000E+01, NA, 4.00000E+00, 1.70000E+01, 1.00000E+00, 0.00000E+00, NA, NA, NA, NA, NA, NA, 6.00000E+00, 3.00000E+00, 2.00000E+00, 5.00000E+00, NA, 2.60000E+01, NA, 4.00000E+00, 1.40000E+01, 2.00000E+00, 2.00000E+00, NA, NA, NA, NA, NA, NA, 9.00000E+00, 2.00000E+00, 4.00000E+00, NA, NA, NA, NA, NA, NA, NA, NA, NA, NA, NA, NA, NA, NA, NA, NA, NA, NA, NA, NA, NA, NA, NA, NA, NA, NA, NA, NA, NA, NA, NA, NA, NA, NA, NA, NA, NA, NA, NA, NA, NA, NA, NA, NA, NA, NA, NA, NA, NA, NA, NA, NA, NA, NA, NA, NA, NA, NA, NA, NA, NA, NA, NA, NA, NA, NA, NA, NA, NA, NA, NA, NA, NA, NA, NA, NA, NA, NA, NA, NA, NA, NA, NA, NA, NA, NA, NA, NA, NA, NA, NA, NA, NA, NA, NA, NA, NA, NA, NA, NA, NA, NA, NA, NA, NA, NA, NA, NA, NA, NA, NA, NA, NA, NA, NA, NA, NA, NA, NA, NA, NA, NA, NA, NA, NA, NA, NA, NA, NA, NA, NA, NA, NA, NA, NA, NA, NA, 1.00000E+01, NA, NA, NA, NA, NA, NA, 2.00000E+00, NA, NA, NA, NA, NA, 0.00000E+00, NA, 0.00000E+00, NA, 1.90000E+01, NA, NA, NA, NA, NA, NA, 5.00000E+00, NA, NA, NA, NA, NA, 0.00000E+00, NA, 1.00000E+00, NA, NA, NA, NA, NA, NA, NA, NA, NA, NA, NA, NA, NA, NA, NA, NA, NA, NA, NA, NA, NA, NA, NA, NA, NA, NA, NA, NA, NA, NA, NA, NA, NA, NA, NA, NA, NA, NA, NA, NA, NA, NA, NA, NA, NA, NA, NA, NA, NA, NA, NA, NA, NA, NA, NA, NA, NA, NA, NA, NA, NA, NA, NA, NA, NA, NA, NA, NA, NA, NA, NA, NA, NA, NA, NA, NA, NA, NA, NA, NA, NA, NA, NA, NA, NA, NA, NA, NA, NA, NA, NA, NA, NA, NA, NA, NA, NA, NA, NA, NA, NA, NA, NA, NA, NA, NA, NA, NA, NA, NA, NA, NA, NA, NA, NA, NA, NA, NA, NA, NA, NA, NA, NA, NA, NA, NA, NA, NA, NA, NA, NA, NA, NA, NA, NA, NA, NA, 3.00000E+00, 1.70000E+01, NA, NA, NA, NA, NA, 5.00000E+00, NA, NA, NA, NA, NA, 0.00000E+00, NA, 5.00000E+00, NA, 1.60000E+01, 1.60000E+01, NA, NA, NA, NA, NA, 0.00000E+00, NA, NA, NA, NA, NA, 0.00000E+00, NA, 0.00000E+00, NA, 4.00000E+00, 1.50000E+01, NA, NA, NA, NA, NA, 6.00000E+00, NA, NA, NA, NA, NA, 0.00000E+00, NA, 6.00000E+00, NA, NA, NA, NA, NA, NA, NA, NA, NA, NA, NA, NA, NA, NA, NA, NA, NA, NA, NA, NA, NA, NA, NA, NA, NA, NA, NA, NA, NA, NA, NA, NA, NA, NA, NA, NA, NA, NA, NA, NA, NA, NA, NA, NA, NA, NA, NA, NA, NA, NA, NA, NA, NA, NA, NA, NA, NA, NA, NA, NA, NA, NA, NA, NA, NA, NA, NA, NA, NA, NA, NA, NA, NA, NA, NA, NA, NA, NA, NA, NA, NA, NA, NA, NA, NA, NA, NA, NA, NA, NA, NA, NA, NA, NA, NA, NA, NA, NA, NA, NA, NA, NA, NA, NA, NA, NA, NA, NA, NA, NA, NA, NA, NA, NA, NA, NA, NA, NA, NA, NA, 2.40000E+01, NA, NA, NA, NA, NA, NA, 2.00000E+00, NA, NA, NA, NA, NA, 0.00000E+00, NA, 2.00000E+00, NA, 2.70000E+01, NA, NA, NA, NA, NA, NA, 7.00000E+00, NA, NA, NA, NA, NA, 1.00000E+00, NA, 7.00000E+00, NA, 3.40000E+01, NA, NA, NA, NA, NA, NA, 7.00000E+00, NA, NA, NA, NA, NA, 0.00000E+00, NA, 7.00000E+00, NA, NA, NA, NA, NA, NA, NA, NA, NA, NA, NA, NA, NA, NA, NA, NA, NA, NA, NA, NA, NA, NA, NA, NA, NA, NA, NA, NA, NA, NA, NA, NA, NA, NA, NA, NA, NA, NA, NA, NA, NA, NA, NA, NA, NA, NA, NA, NA, NA, NA, NA, NA, NA, NA, NA, NA, NA, NA, NA, NA, NA, NA, NA, NA, NA, NA, NA, NA, NA, NA, NA, NA, NA, NA, NA, NA, NA, NA, NA, NA, NA, NA, NA, NA, NA, NA, NA, NA, NA, NA, NA, NA, NA, NA, NA, NA, NA, NA, NA, NA, NA, NA, NA, NA, NA, NA, NA, NA, NA, NA, NA, NA, NA, NA, NA, NA, NA, NA, NA, NA, 7.00000E+00, 1.20000E+01, NA, 0.00000E+00, NA, NA, NA, 8.00000E+00, 1.00000E+00, 2.00000E+00, NA, NA, NA, 4.00000E+00, NA, 8.00000E+00, NA, 1.40000E+01, 1.20000E+01, NA, 1.00000E+00, NA, NA, NA, 1.90000E+01, 2.00000E+00, 4.00000E+00, NA, NA, NA, 3.00000E+00, NA, 1.90000E+01, NA, 7.00000E+00, 6.00000E+00, NA, 2.00000E+00, NA, NA, NA, 7.00000E+00, 2.00000E+00, 2.00000E+00, NA, NA, NA, 1.00000E+00, NA, 7.00000E+00, NA, 1.30000E+01, 1.40000E+01, NA, 0.00000E+00, NA, NA, NA, 1.90000E+01, 0.00000E+00, 0.00000E+00, NA, NA, NA, 2.00000E+00, NA, 1.90000E+01, NA, 8.00000E+00, 8.00000E+00, NA, 2.00000E+00, NA, NA, NA, 9.00000E+00, 7.00000E+00, 0.00000E+00, NA, NA, NA, 1.00000E+00, NA, 9.00000E+00, NA, NA, NA, NA, NA, NA, NA, NA, NA, NA, NA, NA, NA, NA, NA, NA, NA, NA, NA, NA, NA, NA, NA, NA, NA, NA, NA, NA, NA, NA, NA, NA, NA, NA, NA, NA, NA, NA, NA, NA, NA, NA, NA, NA, NA, NA, NA, NA, NA, NA, NA, NA, NA, NA, NA, NA, NA, NA, NA, NA, NA, NA, NA, NA, NA, NA, NA, NA, NA, NA, NA, NA, NA, NA, NA, NA, NA, NA, NA, NA, NA, NA, NA, NA, NA, 1.00000E+00, NA, 2.10000E+01, NA, NA, 5.00000E+00, 5.00000E+00, NA, NA, NA, NA, NA, NA, NA, NA, 1.00000E+00, 7.00000E+00, 8.00000E+00, NA, 4.00000E+00, NA, NA, 6.00000E+00, 8.00000E+00, NA, NA, NA, NA, NA, NA, NA, NA, 0.00000E+00, 1.00000E+00, NA, NA, NA, NA, NA, NA, NA, NA, NA, NA, NA, NA, NA, NA, NA, NA, NA, NA, NA, NA, NA, NA, NA, NA, NA, NA, NA, NA, NA, NA, NA, NA, NA, NA, NA, NA, NA, NA, NA, NA, NA, NA, NA, NA, NA, NA, NA, NA, NA, NA, NA, NA, NA, NA, NA, NA, NA, NA, NA, NA, NA, NA, NA, NA, NA, NA, NA, NA, NA, NA, NA, NA, NA, NA, NA, NA, NA, NA, NA, NA, NA, NA, NA, NA, NA, NA, NA, NA, NA, NA, NA, NA, NA, NA, NA, NA, NA, NA, NA, NA, NA, NA, NA, NA, NA, NA, NA, NA, NA, NA, NA, NA, NA, NA, NA, NA, NA, NA, NA, NA, NA, NA, NA, NA, NA, NA, NA, NA, NA, NA, NA, NA, NA, NA, NA, NA, 1.75000E+02, 2.59800E+03, NA, NA, 1.02000E+02, 6.69000E+02, NA, NA, 8.77000E+02, NA, NA, NA, NA, NA, 1.50000E+01, 2.00000E+02, 3.40000E+02, 1.62000E+02, 2.02900E+03, NA, NA, 9.00000E+01, 6.03000E+02, NA, NA, 6.13000E+02, NA, NA, NA, NA, NA, 1.30000E+01, 9.20000E+01, 2.75000E+02, NA, NA, NA, NA, NA, NA, NA, NA, NA, NA, NA, NA, NA, NA, NA, NA, NA, NA, NA, NA, NA, NA, NA, NA, NA, NA, NA, NA, NA, NA, NA, NA, NA, NA, NA, NA, NA, NA, NA, NA, NA, NA, NA, NA, NA, NA, NA, NA, NA, NA, NA, NA, NA, NA, NA, NA, NA, NA, NA, NA, NA, NA, NA, NA, NA, NA, NA, NA, NA, NA, NA, NA, NA, NA, NA, NA, NA, NA, NA, NA, NA, NA, NA, NA, NA, NA, NA, NA, NA, NA, NA, NA, NA, NA, NA, NA, NA, NA, NA, NA, NA, NA, NA, NA, NA, NA, NA, NA, NA, NA, NA, NA, NA, NA, NA, NA, NA, NA, NA, NA, NA, NA, NA, NA, NA, NA, NA, NA, NA, NA, NA, NA, NA, NA, NA, NA, 1.00000E+00, 3.00000E+00, 1.00000E+01, NA, 0.00000E+00, 0.00000E+00, 1.00000E+00, NA, 4.00000E+00, NA, NA, NA, NA, NA, 3.00000E+00, NA, 4.00000E+00, 0.00000E+00, 1.00000E+00, 8.00000E+00, NA, 0.00000E+00, 0.00000E+00, 0.00000E+00, NA, 1.00000E+00, NA, NA, NA, NA, NA, 0.00000E+00, NA, 1.00000E+00, 0.00000E+00, 1.80000E+01, 1.80000E+01, NA, 0.00000E+00, 0.00000E+00, 0.00000E+00, NA, 1.00000E+00, NA, NA, NA, NA, NA, 0.00000E+00, NA, 1.00000E+00, NA, NA, NA, NA, NA, NA, NA, NA, NA, NA, NA, NA, NA, NA, NA, NA, NA, NA, NA, NA, NA, NA, NA, NA, NA, NA, NA, NA, NA, NA, NA, NA, NA, NA, NA, NA, NA, NA, NA, NA, NA, NA, NA, NA, NA, NA, NA, NA, NA, NA, NA, NA, NA, NA, NA, NA, NA, NA, NA, NA, NA, NA, NA, NA, NA, NA, NA, NA, NA, NA, NA, NA, NA, NA, NA, NA, NA, NA, NA, NA, NA, NA, NA, NA, NA, NA, NA, NA, NA, NA, NA, NA, NA, NA, NA, NA, NA, NA, NA, NA, NA, NA, NA, NA, NA, NA, NA, NA, NA, NA, NA, NA, NA, NA, NA, NA, NA, NA, NA, 3.50000E+01, NA, 1.88000E+02, 4.00000E+00, 2.40000E+01, 1.11000E+02, NA, NA, 1.40000E+02, NA, 8.40000E+01, NA, NA, NA, 4.30000E+01, 1.70000E+01, 1.29000E+02, 9.30000E+01, NA, 1.53000E+02, 6.00000E+00, 2.80000E+01, 1.40000E+02, NA, NA, 1.23000E+02, NA, 9.60000E+01, NA, NA, NA, 1.10000E+02, 2.20000E+01, 1.10000E+02, NA, NA, NA, NA, NA, NA, NA, NA, NA, NA, NA, NA, NA, NA, NA, NA, NA, NA, NA, NA, NA, NA, NA, NA, NA, NA, NA, NA, NA, NA, NA, NA, NA, NA, NA, NA, NA, NA, NA, NA, NA, NA, NA, NA, NA, NA, NA, NA, NA, NA, NA, NA, NA, NA, NA, NA, NA, NA, NA, NA, NA, NA, NA, NA, NA, NA, NA, NA, NA, NA, NA, NA, NA, NA, NA, NA, NA, NA, NA, NA, NA, NA, NA, NA, NA, NA, NA, NA, NA, NA, NA, NA, NA, NA, NA, NA, NA, NA, NA, NA, NA, NA, NA, NA, NA, NA, NA, NA, NA, NA, NA, NA, NA, NA, NA, NA, NA, NA, NA, NA, NA, NA, NA, NA, NA, NA, NA, NA, NA, NA, NA, NA, NA, NA, NA, NA, NA, NA, NA, NA, 1.50000E+01, 1.07000E+02, NA, NA, NA, NA, NA, NA, NA, NA, NA, NA, 2.50000E+01, NA, NA, NA, NA, 1.50000E+01, 1.08000E+02, NA, NA, NA, NA, NA, NA, NA, NA, NA, NA, 2.50000E+01, NA, NA, NA, NA, NA, NA, NA, NA, NA, NA, NA, NA, NA, NA, NA, NA, NA, NA, NA, NA, NA, NA, NA, NA, NA, NA, NA, NA, NA, NA, NA, NA, NA, NA, NA, NA, NA, NA, NA, NA, NA, NA, NA, NA, NA, NA, NA, NA, NA, NA, NA, NA, NA, NA, NA, NA, NA, NA, NA, NA, NA, NA, NA, NA, NA, NA, NA, NA, NA, NA, NA, NA, NA, NA, NA, NA, NA, NA, NA, NA, NA, NA, NA, NA, NA, NA, NA, NA, NA, NA, NA, NA, NA, NA, NA, NA, NA, NA, NA, NA, NA, NA, NA, NA, NA, NA, NA, NA, NA, NA, NA, NA, NA, NA, NA, NA, NA, NA, NA, NA, NA, NA, NA, NA, NA, NA, NA, NA, NA, NA, NA, NA, NA, NA, NA, NA, 2.35000E+02, NA, 7.14000E+02, 5.90000E+01, 1.41000E+02, 8.39000E+02, NA, 8.60000E+01, 1.76100E+03, NA, 6.11000E+02, NA, NA, NA, 2.00000E+01, 2.22000E+02, 1.78800E+03, 3.33000E+02, NA, 5.33000E+02, 2.10000E+01, 1.69000E+02, 7.37000E+02, NA, 7.30000E+01, 1.16100E+03, NA, 5.27000E+02, NA, NA, NA, 2.30000E+01, 7.10000E+01, 1.18200E+03, 2.36000E+02, NA, 6.04000E+02, 3.20000E+01, 1.33000E+02, 7.73000E+02, NA, 8.00000E+01, 1.52800E+03, NA, 5.30000E+02, NA, NA, NA, 1.50000E+01, 1.10000E+02, 1.57100E+03, NA, NA, NA, NA, NA, NA, NA, NA, NA, NA, NA, NA, NA, NA, NA, NA, NA, NA, NA, NA, NA, NA, NA, NA, NA, NA, NA, NA, NA, NA, NA, NA, NA, NA, NA, NA, NA, NA, NA, NA, NA, NA, NA, NA, NA, NA, NA, NA, NA, NA, NA, NA, NA, NA, NA, NA, NA, NA, NA, NA, NA, NA, NA, NA, NA, NA, NA, NA, NA, NA, NA, NA, NA, NA, NA, NA, NA, NA, NA, NA, NA, NA, NA, NA, NA, NA, NA, NA, NA, NA, NA, NA, NA, NA, NA, NA, NA, NA, NA, NA, NA, NA, NA, NA, NA, NA, NA, NA, NA, NA, NA, NA, NA, NA, NA, NA, NA, NA, NA, 0.00000E+00, 4.00000E+01, 3.60000E+01, NA, 0.00000E+00, 1.00000E+00, NA, NA, 7.00000E+00, NA, NA, NA, NA, NA, 0.00000E+00, NA, 9.00000E+00, 0.00000E+00, 2.20000E+01, 2.20000E+01, NA, 0.00000E+00, 1.00000E+00, NA, NA, 1.00000E+00, NA, NA, NA, NA, NA, 0.00000E+00, NA, 1.00000E+00, 1.00000E+00, 4.00000E+00, 2.80000E+01, NA, 0.00000E+00, 0.00000E+00, NA, NA, 5.00000E+00, NA, NA, NA, NA, NA, NA, NA, 5.00000E+00, 1.00000E+00, 2.40000E+01, 2.30000E+01, NA, 0.00000E+00, 0.00000E+00, NA, NA, 5.00000E+00, NA, NA, NA, NA, NA, NA, NA, 5.00000E+00, NA, NA, NA, NA, NA, NA, NA, NA, NA, NA, NA, NA, NA, NA, NA, NA, NA, NA, NA, NA, NA, NA, NA, NA, NA, NA, NA, NA, NA, NA, NA, NA, NA, NA, NA, NA, NA, NA, NA, NA, NA, NA, NA, NA, NA, NA, NA, NA, NA, NA, NA, NA, NA, NA, NA, NA, NA, NA, NA, NA, NA, NA, NA, NA, NA, NA, NA, NA, NA, NA, NA, NA, NA, NA, NA, NA, NA, NA, NA, NA, NA, NA, NA, NA, NA, NA, NA, NA, NA, NA, NA, NA, NA, NA, NA, NA, NA, NA, NA, NA, NA, NA, 3.00000E+00, NA, NA, NA, 3.00000E+00, 1.20000E+01, NA, NA, NA, NA, 9.00000E+00, 3.00000E+00, NA, NA, NA, NA, NA, 4.00000E+00, NA, NA, NA, 2.00000E+00, 1.70000E+01, NA, NA, NA, NA, 9.00000E+00, 5.00000E+00, NA, NA, NA, NA, NA, NA, NA, NA, NA, NA, NA, NA, NA, NA, NA, NA, NA, NA, NA, NA, NA, NA, NA, NA, NA, NA, NA, NA, NA, NA, NA, NA, NA, NA, NA, NA, NA, NA, NA, NA, NA, NA, NA, NA, NA, NA, NA, NA, NA, NA, NA, NA, NA, NA, NA, NA, NA, NA, NA, NA, NA, NA, NA, NA, NA, NA, NA, NA, NA, NA, NA, NA, NA, NA, NA, NA, NA, NA, NA, NA, NA, NA, NA, NA, NA, NA, NA, NA, NA, NA, NA, NA, NA, NA, NA, NA, NA, NA, NA, NA, NA, NA, NA, NA, NA, NA, NA, NA, NA, NA, NA, NA, NA, NA, NA, NA, NA, NA, NA, NA, NA, NA, NA, NA, NA, NA, NA, NA, NA, NA, NA, NA, NA, NA, NA, NA, NA, NA, NA, NA, NA, NA, 1.20000E+01, NA, NA, NA, NA, NA, NA, 4.00000E+00, NA, NA, NA, NA, NA, 0.00000E+00, NA, 0.00000E+00, NA, 1.50000E+01, NA, NA, NA, NA, NA, NA, 9.00000E+00, NA, NA, NA, NA, NA, 0.00000E+00, NA, 0.00000E+00, NA, 7.00000E+00, NA, NA, NA, NA, NA, NA, 2.00000E+00, NA, NA, NA, NA, NA, 0.00000E+00, NA, 0.00000E+00, NA, 8.00000E+00, NA, NA, NA, NA, NA, NA, 2.00000E+00, NA, NA, NA, NA, NA, 0.00000E+00, NA, 0.00000E+00, NA, 1.40000E+01, NA, NA, NA, NA, NA, NA, 6.00000E+00, NA, NA, NA, NA, NA, 0.00000E+00, NA, 0.00000E+00, NA, 1.10000E+01, NA, NA, NA, NA, NA, NA, 6.00000E+00, NA, NA, NA, NA, NA, 0.00000E+00, NA, 3.00000E+00, NA, 1.00000E+01, NA, NA, NA, NA, NA, NA, 5.00000E+00, NA, NA, NA, NA, NA, 0.00000E+00, NA, 1.00000E+00, NA, 2.00000E+00, NA, NA, NA, NA, NA, NA, 0.00000E+00, NA, NA, NA, NA, NA, 1.00000E+00, NA, 0.00000E+00, NA, 3.00000E+00, NA, NA, NA, NA, NA, NA, 1.00000E+00, NA, NA, NA, NA, NA, 0.00000E+00, NA, 0.00000E+00, NA, 2.00000E+00, NA, NA, NA, NA, NA, NA, 1.00000E+00, NA, NA, NA, NA, NA, 1.00000E+00, NA, 0.00000E+00, 1.34000E+02, NA, 1.93100E+03, NA, 7.50000E+01, 4.87000E+02, NA, NA, NA, 2.45800E+03, 3.17000E+02, NA, 1.10000E+01, 3.15000E+02, 2.02000E+02, 1.32000E+02, 3.34000E+02, 1.52000E+02, NA, 1.56600E+03, NA, 9.80000E+01, 4.46000E+02, NA, NA, NA, 2.31100E+03, 2.89000E+02, NA, 1.40000E+01, 2.99000E+02, 1.83000E+02, 4.10000E+01, 3.15000E+02, 1.03000E+02, NA, 1.78700E+03, NA, 9.70000E+01, 4.38000E+02, NA, NA, NA, 2.43000E+03, 2.74000E+02, NA, 1.80000E+01, 3.42000E+02, 1.34000E+02, 4.80000E+01, 3.63000E+02, NA, NA, NA, NA, NA, NA, NA, NA, NA, NA, NA, NA, NA, NA, NA, NA, NA, NA, NA, NA, NA, NA, NA, NA, NA, NA, NA, NA, NA, NA, NA, NA, NA, NA, NA, NA, NA, NA, NA, NA, NA, NA, NA, NA, NA, NA, NA, NA, NA, NA, NA, NA, NA, NA, NA, NA, NA, NA, NA, NA, NA, NA, NA, NA, NA, NA, NA, NA, NA, NA, NA, NA, NA, NA, NA, NA, NA, NA, NA, NA, NA, NA, NA, NA, NA, NA, NA, NA, NA, NA, NA, NA, NA, NA, NA, NA, NA, NA, NA, NA, NA, NA, NA, NA, NA, NA, NA, NA, NA, NA, NA, NA, NA, NA, NA, NA, NA, NA, NA, 1.61000E+02, NA, NA, 5.50000E+01, 1.26000E+02, 2.50000E+02, NA, NA, 1.44900E+03, NA, NA, NA, NA, NA, 2.46000E+02, 8.40000E+01, 1.45300E+03, 1.49000E+02, NA, NA, 2.70000E+01, 1.01000E+02, 2.08000E+02, NA, NA, 1.47500E+03, NA, NA, NA, NA, NA, 2.34000E+02, 5.50000E+01, 1.52500E+03, NA, NA, NA, NA, NA, NA, NA, NA, NA, NA, NA, NA, NA, NA, NA, NA, NA, NA, NA, NA, NA, NA, NA, NA, NA, NA, NA, NA, NA, NA, NA, NA, NA, NA, NA, NA, NA, NA, NA, NA, NA, NA, NA, NA, NA, NA, NA, NA, NA, NA, NA, NA, NA, NA, NA, NA, NA, NA, NA, NA, NA, NA, NA, NA, NA, NA, NA, NA, NA, NA, NA, NA, NA, NA, NA, NA, NA, NA, NA, NA, NA, NA, NA, NA, NA, NA, NA, NA, NA, NA, NA, NA, NA, NA, NA, NA, NA, NA, NA, NA, NA, NA, NA, NA, NA, NA, NA, NA, NA, NA, NA, NA, NA, NA, NA, NA, NA, NA, NA, NA, NA, NA, NA, NA, NA, NA, NA, NA, NA, NA, NA, NA, NA, NA, NA, NA, 1.30000E+01, NA, NA, NA, 1.00000E+01, 3.60000E+01, 7.00000E+00, NA, NA, NA, NA, NA, NA, NA, 1.40000E+01, 6.00000E+00, NA, 1.30000E+01, NA, NA, NA, 5.00000E+00, 2.60000E+01, 3.00000E+00, NA, NA, NA, NA, NA, NA, NA, 1.40000E+01, 7.00000E+00, NA, 1.90000E+01, NA, NA, NA, 1.40000E+01, 4.10000E+01, 1.10000E+01, NA, NA, NA, NA, NA, NA, NA, 2.10000E+01, 2.00000E+00, NA, 1.80000E+01, NA, NA, NA, 5.00000E+00, 2.40000E+01, 4.00000E+00, NA, NA, NA, NA, NA, NA, NA, 1.80000E+01, 3.00000E+00, NA, NA, NA, NA, NA, NA, NA, NA, NA, NA, NA, NA, NA, NA, NA, NA, NA, NA, NA, NA, NA, NA, NA, NA, NA, NA, NA, NA, NA, NA, NA, NA, NA, NA, NA, NA, NA, NA, NA, NA, NA, NA, NA, NA, NA, NA, NA, NA, NA, NA, NA, NA, NA, NA, NA, NA, NA, NA, NA, NA, NA, NA, NA, NA, NA, NA, NA, NA, NA, NA, NA, NA, NA, NA, NA, NA, NA, NA, NA, NA, NA, NA, NA, NA, NA, NA, NA, NA, NA, NA, NA, NA, NA, NA, NA, NA, NA, NA, NA, NA, NA, NA, NA, NA, NA, 6.00000E+00, NA, NA, 1.00000E+00, 0.00000E+00, NA, NA, NA, NA, NA, NA, NA, 0.00000E+00, NA, 0.00000E+00, NA, NA, 4.00000E+00, NA, NA, 2.00000E+00, 1.00000E+00, NA, NA, NA, NA, NA, NA, NA, 0.00000E+00, NA, 3.00000E+00, NA, NA, NA, NA, NA, NA, NA, NA, NA, NA, NA, NA, NA, NA, NA, NA, NA, NA, NA, NA, NA, NA, NA, NA, NA, NA, NA, NA, NA, NA, NA, NA, NA, NA, NA, NA, NA, NA, NA, NA, NA, NA, NA, NA, NA, NA, NA, NA, NA, NA, NA, NA, NA, NA, NA, NA, NA, NA, NA, NA, NA, NA, NA, NA, NA, NA, NA, NA, NA, NA, NA, NA, NA, NA, NA, NA, NA, NA, NA, NA, NA, NA, NA, NA, NA, NA, NA, NA, NA, NA, NA, NA, NA, NA, NA, NA, NA, NA, NA, NA, NA, NA, NA, NA, NA, NA, NA, NA, NA, NA, NA, NA, NA, NA, NA, NA, NA, NA, NA, NA, NA, NA, NA, NA, NA, NA, NA, NA, NA, NA, NA, NA, NA, NA, NA, NA), .Dim=c(20, 10, 17)), E= structure(.Data= c(4.31488E+03, 4.26880E+03, NA, NA, NA, NA, NA, NA, NA, NA, 6.72000E+02, 6.70000E+02, NA, NA, NA, NA, NA, NA, NA, NA, 3.55000E+02, 3.65000E+02, NA, NA, NA, NA, NA, NA, NA, NA, 1.06154E+01, 1.33846E+01, NA, NA, NA, NA, NA, NA, NA, NA, 1.87500E+01, 1.97500E+01, 2.00000E+01, NA, NA, NA, NA, NA, NA, NA, 3.00000E+01, 3.09231E+01, 3.00000E+01, NA, NA, NA, NA, NA, NA, NA, 5.76923E+01, 5.63077E+01, 5.42308E+01, 4.15385E+01, 5.40000E+01, NA, NA, NA, NA, NA, 2.98750E+02, 2.51250E+02, NA, NA, NA, NA, NA, NA, NA, NA, 1.63458E+04, 1.64160E+04, NA, NA, NA, NA, NA, NA, NA, NA, 1.73077E+01, 1.66154E+01, 1.63846E+01, NA, NA, NA, NA, NA, NA, NA, 3.08880E+03, 3.07010E+03, NA, NA, NA, NA, NA, NA, NA, NA, 1.31760E+03, 1.30950E+03, NA, NA, NA, NA, NA, NA, NA, NA, 1.87847E+04, 1.87793E+04, 1.87820E+04, NA, NA, NA, NA, NA, NA, NA, 5.11479E+01, 5.11479E+01, 5.11479E+01, 5.11479E+01, NA, NA, NA, NA, NA, NA, 3.48435E+02, 3.75033E+02, NA, NA, NA, NA, NA, NA, NA, NA, 1.61538E+01, 2.30769E+01, 7.61538E+00, 8.30769E+00, 2.42308E+01, 6.92308E+00, 7.84615E+00, 1.36154E+01, 6.23077E+00, 4.84615E+00, 1.20440E+04, 1.20300E+04, 1.21520E+04, NA, NA, NA, NA, NA, NA, NA, 1.38165E+04, 1.38126E+04, NA, NA, NA, NA, NA, NA, NA, NA, 1.09900E+03, 3.94000E+02, 1.08300E+03, 3.77000E+02, NA, NA, NA, NA, NA, NA, 3.60000E+01, 3.90000E+01, NA, NA, NA, NA, NA, NA, NA, NA), .Dim=c(20, 10)), E.observed= structure(.Data= c(1.00000E+00, 1.00000E+00, NA, NA, NA, NA, NA, NA, NA, NA, 0.00000E+00, 0.00000E+00, NA, NA, NA, NA, NA, NA, NA, NA, 1.00000E+00, 1.00000E+00, NA, NA, NA, NA, NA, NA, NA, NA, 0.00000E+00, 0.00000E+00, NA, NA, NA, NA, NA, NA, NA, NA, 0.00000E+00, 0.00000E+00, 0.00000E+00, NA, NA, NA, NA, NA, NA, NA, 0.00000E+00, 0.00000E+00, 0.00000E+00, NA, NA, NA, NA, NA, NA, NA, 0.00000E+00, 0.00000E+00, 0.00000E+00, 0.00000E+00, 0.00000E+00, NA, NA, NA, NA, NA, 1.00000E+00, 1.00000E+00, NA, NA, NA, NA, NA, NA, NA, NA, 1.00000E+00, 1.00000E+00, NA, NA, NA, NA, NA, NA, NA, NA, 0.00000E+00, 0.00000E+00, 0.00000E+00, NA, NA, NA, NA, NA, NA, NA, 1.00000E+00, 1.00000E+00, NA, NA, NA, NA, NA, NA, NA, NA, 1.00000E+00, 1.00000E+00, NA, NA, NA, NA, NA, NA, NA, NA, 1.00000E+00, 1.00000E+00, 1.00000E+00, NA, NA, NA, NA, NA, NA, NA, 1.00000E+00, 1.00000E+00, 1.00000E+00, 1.00000E+00, NA, NA, NA, NA, NA, NA, 1.00000E+00, 1.00000E+00, NA, NA, NA, NA, NA, NA, NA, NA, 0.00000E+00, 0.00000E+00, 0.00000E+00, 0.00000E+00, 0.00000E+00, 0.00000E+00, 0.00000E+00, 0.00000E+00, 0.00000E+00, 0.00000E+00, 1.00000E+00, 1.00000E+00, 1.00000E+00, NA, NA, NA, NA, NA, NA, NA, 1.00000E+00, 1.00000E+00, NA, NA, NA, NA, NA, NA, NA, NA, 1.00000E+00, 1.00000E+00, 1.00000E+00, 1.00000E+00, NA, NA, NA, NA, NA, NA, 0.00000E+00, 0.00000E+00, NA, NA, NA, NA, NA, NA, NA, NA), .Dim=c(20, 10)), na=c(2.00000E+00, 2.00000E+00, 2.00000E+00, 2.00000E+00, 3.00000E+00, 3.00000E+00, 5.00000E+00, 2.00000E+00, 2.00000E+00, 3.00000E+00, 2.00000E+00, 2.00000E+00, 3.00000E+00, 4.00000E+00, 2.00000E+00, 1.00000E+01, 3.00000E+00, 2.00000E+00, 4.00000E+00, 2.00000E+00), t= structure(.Data= c(1.00000E+00, 2.00000E+00, NA, NA, NA, NA, NA, NA, NA, NA, 8.00000E+00, 9.00000E+00, NA, NA, NA, NA, NA, NA, NA, NA, 1.00000E+00, 2.00000E+00, NA, NA, NA, NA, NA, NA, NA, NA, 1.00000E+01, 1.10000E+01, NA, NA, NA, NA, NA, NA, NA, NA, 1.00000E+00, 2.10000E+01, 2.40000E+01, NA, NA, NA, NA, NA, NA, NA, 2.10000E+01, 2.20000E+01, 2.40000E+01, NA, NA, NA, NA, NA, NA, NA, 1.00000E+00, 2.00000E+01, 2.10000E+01, 2.30000E+01, 2.40000E+01, NA, NA, NA, NA, NA, 1.00000E+00, 2.00000E+00, NA, NA, NA, NA, NA, NA, NA, NA, 1.00000E+00, 4.00000E+00, NA, NA, NA, NA, NA, NA, NA, NA, 1.00000E+00, 3.00000E+00, 4.00000E+00, NA, NA, NA, NA, NA, NA, NA, 4.00000E+00, 8.00000E+00, NA, NA, NA, NA, NA, NA, NA, NA, 1.00000E+00, 8.00000E+00, NA, NA, NA, NA, NA, NA, NA, NA, 1.00000E+00, 2.10000E+01, 2.40000E+01, NA, NA, NA, NA, NA, NA, NA, 1.00000E+00, 5.00000E+00, 6.00000E+00, 7.00000E+00, NA, NA, NA, NA, NA, NA, 1.00000E+00, 2.00000E+00, NA, NA, NA, NA, NA, NA, NA, NA, 1.00000E+00, 1.10000E+01, 1.20000E+01, 1.30000E+01, 1.40000E+01, 1.50000E+01, 1.60000E+01, 1.70000E+01, 1.80000E+01, 1.90000E+01, 1.00000E+00, 1.00000E+01, 1.10000E+01, NA, NA, NA, NA, NA, NA, NA, 1.00000E+00, 2.50000E+01, NA, NA, NA, NA, NA, NA, NA, NA, 1.00000E+00, 1.00000E+00, 2.00000E+00, 2.00000E+00, NA, NA, NA, NA, NA, NA, 1.00000E+00, 2.00000E+00, NA, NA, NA, NA, NA, NA, NA, NA), .Dim=c(20, 10)), n= structure(.Data= c(3.37100E+03, 3.33500E+03, NA, NA, NA, NA, NA, NA, NA, NA, 3.36000E+02, 3.35000E+02, NA, NA, NA, NA, NA, NA, NA, NA, 1.70000E+02, 1.69000E+02, NA, NA, NA, NA, NA, NA, NA, NA, 4.60000E+01, 5.80000E+01, NA, NA, NA, NA, NA, NA, NA, NA, 7.50000E+01, 7.90000E+01, 8.00000E+01, NA, NA, NA, NA, NA, NA, NA, 1.30000E+02, 1.34000E+02, 1.30000E+02, NA, NA, NA, NA, NA, NA, NA, 2.50000E+02, 2.44000E+02, 2.35000E+02, 1.80000E+02, 2.34000E+02, NA, NA, NA, NA, NA, 2.39000E+02, 2.01000E+02, NA, NA, NA, NA, NA, NA, NA, NA, 9.08100E+03, 9.12000E+03, NA, NA, NA, NA, NA, NA, NA, NA, 7.50000E+01, 7.20000E+01, 7.10000E+01, NA, NA, NA, NA, NA, NA, NA, 2.80800E+03, 2.79100E+03, NA, NA, NA, NA, NA, NA, NA, NA, 4.88000E+02, 4.85000E+02, NA, NA, NA, NA, NA, NA, NA, NA, 7.03600E+03, 7.03400E+03, 7.03500E+03, NA, NA, NA, NA, NA, NA, NA, 1.27000E+02, 1.27000E+02, 1.27000E+02, 1.27000E+02, NA, NA, NA, NA, NA, NA, 1.31000E+02, 1.41000E+02, NA, NA, NA, NA, NA, NA, NA, NA, 7.00000E+01, 1.00000E+02, 3.30000E+01, 3.60000E+01, 1.05000E+02, 3.00000E+01, 3.40000E+01, 5.90000E+01, 2.70000E+01, 2.10000E+01, 6.02200E+03, 6.01500E+03, 6.07600E+03, NA, NA, NA, NA, NA, NA, NA, 7.08200E+03, 7.06100E+03, NA, NA, NA, NA, NA, NA, NA, NA, 3.58000E+02, 1.97000E+02, 3.57000E+02, 1.88000E+02, NA, NA, NA, NA, NA, NA, 3.60000E+01, 3.90000E+01, NA, NA, NA, NA, NA, NA, NA, NA), .Dim=c(20, 10)), f= structure(.Data= c(1.28000E+00, 1.28000E+00, NA, NA, NA, NA, NA, NA, NA, NA, 2.00000E+00, 2.00000E+00, NA, NA, NA, NA, NA, NA, NA, NA, 2.08824E+00, 2.15976E+00, NA, NA, NA, NA, NA, NA, NA, NA, 2.30769E-01, 2.30769E-01, NA, NA, NA, NA, NA, NA, NA, NA, 2.50000E-01, 2.50000E-01, 2.50000E-01, NA, NA, NA, NA, NA, NA, NA, 2.30769E-01, 2.30769E-01, 2.30769E-01, NA, NA, NA, NA, NA, NA, NA, 2.30769E-01, 2.30769E-01, 2.30769E-01, 2.30769E-01, 2.30769E-01, NA, NA, NA, NA, NA, 1.25000E+00, 1.25000E+00, NA, NA, NA, NA, NA, NA, NA, NA, 1.80000E+00, 1.80000E+00, NA, NA, NA, NA, NA, NA, NA, NA, 2.30769E-01, 2.30769E-01, 2.30769E-01, NA, NA, NA, NA, NA, NA, NA, 1.10000E+00, 1.10000E+00, NA, NA, NA, NA, NA, NA, NA, NA, 2.70000E+00, 2.70000E+00, NA, NA, NA, NA, NA, NA, NA, NA, 2.66979E+00, 2.66979E+00, 2.66979E+00, NA, NA, NA, NA, NA, NA, NA, 4.02740E-01, 4.02740E-01, 4.02740E-01, 4.02740E-01, NA, NA, NA, NA, NA, NA, 2.65981E+00, 2.65981E+00, NA, NA, NA, NA, NA, NA, NA, NA, 2.30769E-01, 2.30769E-01, 2.30769E-01, 2.30769E-01, 2.30769E-01, 2.30769E-01, 2.30769E-01, 2.30769E-01, 2.30769E-01, 2.30769E-01, 2.00000E+00, 2.00000E+00, 2.00000E+00, NA, NA, NA, NA, NA, NA, NA, 1.93699E+00, 1.93699E+00, NA, NA, NA, NA, NA, NA, NA, NA, 3.06983E+00, 2.00000E+00, 3.03361E+00, 2.00532E+00, NA, NA, NA, NA, NA, NA, 1.00000E+00, 1.00000E+00, NA, NA, NA, NA, NA, NA, NA, NA), .Dim=c(20, 10)), f.observed= structure(.Data= c(1.00000E+00, 1.00000E+00, NA, NA, NA, NA, NA, NA, NA, NA, 0.00000E+00, 0.00000E+00, NA, NA, NA, NA, NA, NA, NA, NA, 1.00000E+00, 1.00000E+00, NA, NA, NA, NA, NA, NA, NA, NA, 0.00000E+00, 0.00000E+00, NA, NA, NA, NA, NA, NA, NA, NA, 0.00000E+00, 0.00000E+00, 0.00000E+00, NA, NA, NA, NA, NA, NA, NA, 0.00000E+00, 0.00000E+00, 0.00000E+00, NA, NA, NA, NA, NA, NA, NA, 0.00000E+00, 0.00000E+00, 0.00000E+00, 0.00000E+00, 0.00000E+00, NA, NA, NA, NA, NA, 1.00000E+00, 1.00000E+00, NA, NA, NA, NA, NA, NA, NA, NA, 1.00000E+00, 1.00000E+00, NA, NA, NA, NA, NA, NA, NA, NA, 0.00000E+00, 0.00000E+00, 0.00000E+00, NA, NA, NA, NA, NA, NA, NA, 1.00000E+00, 1.00000E+00, NA, NA, NA, NA, NA, NA, NA, NA, 1.00000E+00, 1.00000E+00, NA, NA, NA, NA, NA, NA, NA, NA, 1.00000E+00, 1.00000E+00, 1.00000E+00, NA, NA, NA, NA, NA, NA, NA, 1.00000E+00, 1.00000E+00, 1.00000E+00, 1.00000E+00, NA, NA, NA, NA, NA, NA, 1.00000E+00, 1.00000E+00, NA, NA, NA, NA, NA, NA, NA, NA, 0.00000E+00, 0.00000E+00, 0.00000E+00, 0.00000E+00, 0.00000E+00, 0.00000E+00, 0.00000E+00, 0.00000E+00, 0.00000E+00, 0.00000E+00, 1.00000E+00, 1.00000E+00, 1.00000E+00, NA, NA, NA, NA, NA, NA, NA, 1.00000E+00, 1.00000E+00, NA, NA, NA, NA, NA, NA, NA, NA, 1.00000E+00, 1.00000E+00, 1.00000E+00, 1.00000E+00, NA, NA, NA, NA, NA, NA, 0.00000E+00, 0.00000E+00, NA, NA, NA, NA, NA, NA, NA, NA), .Dim=c(20, 10)), m=1.70000E+01, active_w.outcomes=c(1.00000E+00, 2.00000E+00, 3.00000E+00, 4.00000E+00, 5.00000E+00, 6.00000E+00, 1.60000E+01, 1.70000E+01), n.active_w.outcomes=8.00000E+00, active_w.j=1.00000E+00, active_w.R=c(1.37800E+03, 1.61000E+03, 0.00000E+00, 0.00000E+00, 0.00000E+00, 0.00000E+00, 0.00000E+00, 0.00000E+00, 0.00000E+00, 0.00000E+00), active_w.r= structure(.Data= c(4.20000E+01, 5.55000E+02, 4.81000E+02, 1.10000E+01, 2.30000E+01, 1.58000E+02, 1.50000E+01, 9.30000E+01, 9.00000E+01, 6.44000E+02, 5.68000E+02, 7.00000E+00, 3.60000E+01, 1.59000E+02, 5.00000E+00, 1.01000E+02, NA, NA, NA, NA, NA, NA, NA, NA, NA, NA, NA, NA, NA, NA, NA, NA, NA, NA, NA, NA, NA, NA, NA, NA, NA, NA, NA, NA, NA, NA, NA, NA, NA, NA, NA, NA, NA, NA, NA, NA, NA, NA, NA, NA, NA, NA, NA, NA, NA, NA, NA, NA, NA, NA, NA, NA, NA, NA, NA, NA, NA, NA, NA, NA), .Dim=c(10, 8)), averroes.outcomes=c(1.00000E+00, 3.00000E+00, 4.00000E+00, 5.00000E+00, 6.00000E+00, 9.00000E+00, 1.10000E+01, 1.50000E+01, 1.60000E+01, 1.70000E+01), n.averroes.outcomes=1.00000E+01, averroes.j=1.10000E+01, averroes.R=c(7.75000E+02, 8.81000E+02, 0.00000E+00, 0.00000E+00, 0.00000E+00, 0.00000E+00, 0.00000E+00, 0.00000E+00, 0.00000E+00, 0.00000E+00), averroes.r= structure(.Data= c(3.50000E+01, 1.88000E+02, 4.00000E+00, 2.40000E+01, 1.11000E+02, 1.40000E+02, 8.40000E+01, 4.30000E+01, 1.70000E+01, 1.29000E+02, 9.30000E+01, 1.53000E+02, 6.00000E+00, 2.80000E+01, 1.40000E+02, 1.23000E+02, 9.60000E+01, 1.10000E+02, 2.20000E+01, 1.10000E+02, NA, NA, NA, NA, NA, NA, NA, NA, NA, NA, NA, NA, NA, NA, NA, NA, NA, NA, NA, NA, NA, NA, NA, NA, NA, NA, NA, NA, NA, NA, NA, NA, NA, NA, NA, NA, NA, NA, NA, NA, NA, NA, NA, NA, NA, NA, NA, NA, NA, NA, NA, NA, NA, NA, NA, NA, NA, NA, NA, NA, NA, NA, NA, NA, NA, NA, NA, NA, NA, NA, NA, NA, NA, NA, NA, NA, NA, NA, NA, NA), .Dim=c(10, 10)), bafta.outcomes=c(5.00000E+00, 6.00000E+00, 1.70000E+01), n.bafta.outcomes=3.00000E+00, bafta.j=1.20000E+01, bafta.R=c(1.47000E+02, 1.48000E+02, 0.00000E+00, 0.00000E+00, 0.00000E+00, 0.00000E+00, 0.00000E+00, 0.00000E+00, 0.00000E+00, 0.00000E+00), bafta.r= structure(.Data= c(1.50000E+01, 1.07000E+02, 2.50000E+01, 1.50000E+01, 1.08000E+02, 2.50000E+01, NA, NA, NA, NA, NA, NA, NA, NA, NA, NA, NA, NA, NA, NA, NA, NA, NA, NA, NA, NA, NA, NA, NA, NA), .Dim=c(10, 3)), pataf.outcomes=c(1.00000E+00, 5.00000E+00, 6.00000E+00, 1.10000E+01, 1.20000E+01), n.pataf.outcomes=5.00000E+00, pataf.j=1.50000E+01, pataf.R=c(3.00000E+01, 3.70000E+01, 0.00000E+00, 0.00000E+00, 0.00000E+00, 0.00000E+00, 0.00000E+00, 0.00000E+00, 0.00000E+00, 0.00000E+00), pataf.r= structure(.Data= c(3.00000E+00, 3.00000E+00, 1.20000E+01, 9.00000E+00, 3.00000E+00, 4.00000E+00, 2.00000E+00, 1.70000E+01, 9.00000E+00, 5.00000E+00, NA, NA, NA, NA, NA, NA, NA, NA, NA, NA, NA, NA, NA, NA, NA, NA, NA, NA, NA, NA, NA, NA, NA, NA, NA, NA, NA, NA, NA, NA, NA, NA, NA, NA, NA, NA, NA, NA, NA, NA), .Dim=c(10, 5)), afasak_ii.outcomes=c(1.00000E+00, 3.00000E+00, 5.00000E+00, 6.00000E+00, 7.00000E+00, 8.00000E+00, 1.50000E+01, 1.60000E+01, 1.70000E+01), n.afasak_ii.outcomes=9.00000E+00, afasak_ii.j=3.00000E+00, afasak_ii.R=c(7.80000E+01, 6.80000E+01, 0.00000E+00, 0.00000E+00, 0.00000E+00, 0.00000E+00, 0.00000E+00, 0.00000E+00, 0.00000E+00, 0.00000E+00), afasak_ii.r= structure(.Data= c(3.00000E+00, 4.20000E+01, 4.00000E+00, 1.70000E+01, 1.00000E+00, 0.00000E+00, 6.00000E+00, 3.00000E+00, 2.00000E+00, 5.00000E+00, 2.60000E+01, 4.00000E+00, 1.40000E+01, 2.00000E+00, 2.00000E+00, 9.00000E+00, 2.00000E+00, 4.00000E+00, NA, NA, NA, NA, NA, NA, NA, NA, NA, NA, NA, NA, NA, NA, NA, NA, NA, NA, NA, NA, NA, NA, NA, NA, NA, NA, NA, NA, NA, NA, NA, NA, NA, NA, NA, NA, NA, NA, NA, NA, NA, NA, NA, NA, NA, NA, NA, NA, NA, NA, NA, NA, NA, NA, NA, NA, NA, NA, NA, NA, NA, NA, NA, NA, NA, NA, NA, NA, NA, NA, NA, NA), .Dim=c(10, 9)), outcomes.to.save=c(1.00000E+00, 5.00000E+00, 6.00000E+00, 1.70000E+01), n.to.save=4.00000E+00)*

*## Initial values for one chain #################################################*

*list(mu= structure(.Data= c(1.00000E-01, 1.00000E-01, 1.00000E-01, 1.00000E-01, 1.00000E-01, 1.00000E-01, 1.00000E-01, 1.00000E-01, 1.00000E-01, 1.00000E-01, 1.00000E-01, 1.00000E-01, 1.00000E-01, 1.00000E-01, 1.00000E-01, 1.00000E-01, 1.00000E-01, 1.00000E-01, 1.00000E-01, 1.00000E-01, 1.00000E-01, 1.00000E-01, 1.00000E-01, 1.00000E-01, 1.00000E-01, 1.00000E-01, 1.00000E-01, 1.00000E-01, 1.00000E-01, 1.00000E-01, 1.00000E-01, 1.00000E-01, 1.00000E-01, 1.00000E-01, 1.00000E-01, 1.00000E-01, 1.00000E-01, 1.00000E-01, 1.00000E-01, 1.00000E-01, 1.00000E-01, 1.00000E-01, 1.00000E-01, 1.00000E-01, 1.00000E-01, 1.00000E-01, 1.00000E-01, 1.00000E-01, 1.00000E-01, 1.00000E-01, 1.00000E-01, 1.00000E-01, 1.00000E-01, 1.00000E-01, 1.00000E-01, 1.00000E-01, 1.00000E-01, 1.00000E-01, 1.00000E-01, 1.00000E-01, 1.00000E-01, 1.00000E-01, 1.00000E-01, 1.00000E-01, 1.00000E-01, 1.00000E-01, 1.00000E-01, 1.00000E-01, 1.00000E-01, 1.00000E-01, 1.00000E-01, 1.00000E-01, 1.00000E-01, 1.00000E-01, 1.00000E-01, 1.00000E-01, 1.00000E-01, 1.00000E-01, 1.00000E-01, 1.00000E-01, 1.00000E-01, 1.00000E-01, 1.00000E-01, 1.00000E-01, 1.00000E-01, 1.00000E-01, 1.00000E-01, 1.00000E-01, 1.00000E-01, 1.00000E-01, 1.00000E-01, 1.00000E-01, 1.00000E-01, 1.00000E-01, 1.00000E-01, 1.00000E-01, 1.00000E-01, 1.00000E-01, 1.00000E-01, 1.00000E-01, 1.00000E-01, 1.00000E-01, 1.00000E-01, 1.00000E-01, 1.00000E-01, 1.00000E-01, 1.00000E-01, 1.00000E-01, 1.00000E-01, 1.00000E-01, 1.00000E-01, 1.00000E-01, 1.00000E-01, 1.00000E-01, 1.00000E-01, 1.00000E-01, 1.00000E-01, 1.00000E-01, 1.00000E-01, 1.00000E-01, 1.00000E-01, 1.00000E-01, 1.00000E-01, 1.00000E-01, 1.00000E-01, 1.00000E-01, 1.00000E-01, 1.00000E-01, 1.00000E-01, 1.00000E-01, 1.00000E-01, 1.00000E-01, 1.00000E-01, 1.00000E-01, 1.00000E-01, 1.00000E-01, 1.00000E-01, 1.00000E-01, 1.00000E-01, 1.00000E-01, 1.00000E-01, 1.00000E-01, 1.00000E-01, 1.00000E-01, 1.00000E-01, 1.00000E-01, 1.00000E-01, 1.00000E-01, 1.00000E-01, 1.00000E-01, 1.00000E-01, 1.00000E-01, 1.00000E-01, 1.00000E-01, 1.00000E-01, 1.00000E-01, 1.00000E-01, 1.00000E-01, 1.00000E-01, 1.00000E-01, 1.00000E-01, 1.00000E-01, 1.00000E-01, 1.00000E-01, 1.00000E-01, 1.00000E-01, 1.00000E-01, 1.00000E-01, 1.00000E-01, 1.00000E-01, 1.00000E-01, 1.00000E-01, 1.00000E-01, 1.00000E-01, 1.00000E-01, 1.00000E-01, 1.00000E-01, 1.00000E-01, 1.00000E-01, 1.00000E-01, 1.00000E-01, 1.00000E-01, 1.00000E-01, 1.00000E-01, 1.00000E-01, 1.00000E-01, 1.00000E-01, 1.00000E-01, 1.00000E-01, 1.00000E-01, 1.00000E-01, 1.00000E-01, 1.00000E-01, 1.00000E-01, 1.00000E-01, 1.00000E-01, 1.00000E-01, 1.00000E-01, 1.00000E-01, 1.00000E-01, 1.00000E-01, 1.00000E-01, 1.00000E-01, 1.00000E-01, 1.00000E-01, 1.00000E-01, 1.00000E-01, 1.00000E-01, 1.00000E-01, 1.00000E-01, 1.00000E-01, 1.00000E-01, 1.00000E-01, 1.00000E-01, 1.00000E-01, 1.00000E-01, 1.00000E-01, 1.00000E-01, 1.00000E-01, 1.00000E-01, 1.00000E-01, 1.00000E-01, 1.00000E-01, 1.00000E-01, 1.00000E-01, 1.00000E-01, 1.00000E-01, 1.00000E-01, 1.00000E-01, 1.00000E-01, 1.00000E-01, 1.00000E-01, 1.00000E-01, 1.00000E-01, 1.00000E-01, 1.00000E-01, 1.00000E-01, 1.00000E-01, 1.00000E-01, 1.00000E-01, 1.00000E-01, 1.00000E-01, 1.00000E-01, 1.00000E-01, 1.00000E-01, 1.00000E-01, 1.00000E-01, 1.00000E-01, 1.00000E-01, 1.00000E-01, 1.00000E-01, 1.00000E-01, 1.00000E-01, 1.00000E-01, 1.00000E-01, 1.00000E-01, 1.00000E-01, 1.00000E-01, 1.00000E-01, 1.00000E-01, 1.00000E-01, 1.00000E-01, 1.00000E-01, 1.00000E-01, 1.00000E-01, 1.00000E-01, 1.00000E-01, 1.00000E-01, 1.00000E-01, 1.00000E-01, 1.00000E-01, 1.00000E-01, 1.00000E-01, 1.00000E-01, 1.00000E-01, 1.00000E-01, 1.00000E-01, 1.00000E-01, 1.00000E-01, 1.00000E-01, 1.00000E-01, 1.00000E-01, 1.00000E-01, 1.00000E-01, 1.00000E-01, 1.00000E-01, 1.00000E-01, 1.00000E-01, 1.00000E-01, 1.00000E-01, 1.00000E-01, 1.00000E-01, 1.00000E-01, 1.00000E-01, 1.00000E-01, 1.00000E-01, 1.00000E-01, 1.00000E-01, 1.00000E-01, 1.00000E-01, 1.00000E-01, 1.00000E-01, 1.00000E-01, 1.00000E-01, 1.00000E-01, 1.00000E-01, 1.00000E-01, 1.00000E-01, 1.00000E-01, 1.00000E-01, 1.00000E-01, 1.00000E-01, 1.00000E-01, 1.00000E-01, 1.00000E-01, 1.00000E-01, 1.00000E-01, 1.00000E-01, 1.00000E-01, 1.00000E-01, 1.00000E-01, 1.00000E-01, 1.00000E-01, 1.00000E-01, 1.00000E-01, 1.00000E-01, 1.00000E-01, 1.00000E-01, 1.00000E-01, 1.00000E-01, 1.00000E-01, 1.00000E-01, 1.00000E-01, 1.00000E-01, 1.00000E-01, 1.00000E-01, 1.00000E-01, 1.00000E-01, 1.00000E-01, 1.00000E-01), .Dim=c(20, 17)), d= structure(.Data= c( NA, NA, NA, NA, NA, NA, NA, NA, NA, NA, NA, NA, NA, NA, NA, NA, NA, 1.00000E-01, 1.00000E-01, 1.00000E-01, 1.00000E-01, 1.00000E-01, 1.00000E-01, 1.00000E-01, 1.00000E-01, 1.00000E-01, 1.00000E-01, 1.00000E-01, 1.00000E-01, 1.00000E-01, 1.00000E-01, 1.00000E-01, 1.00000E-01, 1.00000E-01, 1.00000E-01, 1.00000E-01, 1.00000E-01, 1.00000E-01, 1.00000E-01, 1.00000E-01, 1.00000E-01, 1.00000E-01, 1.00000E-01, 1.00000E-01, 1.00000E-01, 1.00000E-01, 1.00000E-01, 1.00000E-01, 1.00000E-01, 1.00000E-01, 1.00000E-01, 1.00000E-01, 1.00000E-01, 1.00000E-01, 1.00000E-01, 1.00000E-01, 1.00000E-01, 1.00000E-01, 1.00000E-01, 1.00000E-01, 1.00000E-01, 1.00000E-01, 1.00000E-01, 1.00000E-01, 1.00000E-01, 1.00000E-01, 1.00000E-01, 1.00000E-01, 1.00000E-01, 1.00000E-01, 1.00000E-01, 1.00000E-01, 1.00000E-01, 1.00000E-01, 1.00000E-01, 1.00000E-01, 1.00000E-01, 1.00000E-01, 1.00000E-01, 1.00000E-01, 1.00000E-01, 1.00000E-01, 1.00000E-01, 1.00000E-01, 1.00000E-01, 1.00000E-01, 1.00000E-01, 1.00000E-01, 1.00000E-01, 1.00000E-01, 1.00000E-01, 1.00000E-01, 1.00000E-01, 1.00000E-01, 1.00000E-01, 1.00000E-01, 1.00000E-01, 1.00000E-01, 1.00000E-01, 1.00000E-01, 1.00000E-01, 1.00000E-01, 1.00000E-01, 1.00000E-01, 1.00000E-01, 1.00000E-01, 1.00000E-01, 1.00000E-01, 1.00000E-01, 1.00000E-01, 1.00000E-01, 1.00000E-01, 1.00000E-01, 1.00000E-01, 1.00000E-01, 1.00000E-01, 1.00000E-01, 1.00000E-01, 1.00000E-01, 1.00000E-01, 1.00000E-01, 1.00000E-01, 1.00000E-01, 1.00000E-01, 1.00000E-01, 1.00000E-01, 1.00000E-01, 1.00000E-01, 1.00000E-01, 1.00000E-01, 1.00000E-01, 1.00000E-01, 1.00000E-01, 1.00000E-01, 1.00000E-01, 1.00000E-01, 1.00000E-01, 1.00000E-01, 1.00000E-01, 1.00000E-01, 1.00000E-01, 1.00000E-01, 1.00000E-01, 1.00000E-01, 1.00000E-01, 1.00000E-01, 1.00000E-01, 1.00000E-01, 1.00000E-01, 1.00000E-01, 1.00000E-01, 1.00000E-01, 1.00000E-01, 1.00000E-01, 1.00000E-01, 1.00000E-01, 1.00000E-01, 1.00000E-01, 1.00000E-01, 1.00000E-01, 1.00000E-01, 1.00000E-01, 1.00000E-01, 1.00000E-01, 1.00000E-01, 1.00000E-01, 1.00000E-01, 1.00000E-01, 1.00000E-01, 1.00000E-01, 1.00000E-01, 1.00000E-01, 1.00000E-01, 1.00000E-01, 1.00000E-01, 1.00000E-01, 1.00000E-01, 1.00000E-01, 1.00000E-01, 1.00000E-01, 1.00000E-01, 1.00000E-01, 1.00000E-01, 1.00000E-01, 1.00000E-01, 1.00000E-01, 1.00000E-01, 1.00000E-01, 1.00000E-01, 1.00000E-01, 1.00000E-01, 1.00000E-01, 1.00000E-01, 1.00000E-01, 1.00000E-01, 1.00000E-01, 1.00000E-01, 1.00000E-01, 1.00000E-01, 1.00000E-01, 1.00000E-01, 1.00000E-01, 1.00000E-01, 1.00000E-01, 1.00000E-01, 1.00000E-01, 1.00000E-01, 1.00000E-01, 1.00000E-01, 1.00000E-01, 1.00000E-01, 1.00000E-01, 1.00000E-01, 1.00000E-01, 1.00000E-01, 1.00000E-01, 1.00000E-01, 1.00000E-01, 1.00000E-01, 1.00000E-01, 1.00000E-01, 1.00000E-01, 1.00000E-01, 1.00000E-01, 1.00000E-01, 1.00000E-01, 1.00000E-01, 1.00000E-01, 1.00000E-01, 1.00000E-01, 1.00000E-01, 1.00000E-01, 1.00000E-01, 1.00000E-01, 1.00000E-01, 1.00000E-01, 1.00000E-01, 1.00000E-01, 1.00000E-01, 1.00000E-01, 1.00000E-01, 1.00000E-01, 1.00000E-01, 1.00000E-01, 1.00000E-01, 1.00000E-01, 1.00000E-01, 1.00000E-01, 1.00000E-01, 1.00000E-01, 1.00000E-01, 1.00000E-01, 1.00000E-01, 1.00000E-01, 1.00000E-01, 1.00000E-01, 1.00000E-01, 1.00000E-01, 1.00000E-01, 1.00000E-01, 1.00000E-01, 1.00000E-01, 1.00000E-01, 1.00000E-01, 1.00000E-01, 1.00000E-01, 1.00000E-01, 1.00000E-01, 1.00000E-01, 1.00000E-01, 1.00000E-01, 1.00000E-01, 1.00000E-01, 1.00000E-01, 1.00000E-01, 1.00000E-01, 1.00000E-01, 1.00000E-01, 1.00000E-01, 1.00000E-01, 1.00000E-01, 1.00000E-01, 1.00000E-01, 1.00000E-01, 1.00000E-01, 1.00000E-01, 1.00000E-01, 1.00000E-01, 1.00000E-01, 1.00000E-01, 1.00000E-01, 1.00000E-01, 1.00000E-01, 1.00000E-01, 1.00000E-01, 1.00000E-01, 1.00000E-01, 1.00000E-01, 1.00000E-01, 1.00000E-01, 1.00000E-01, 1.00000E-01, 1.00000E-01, 1.00000E-01, 1.00000E-01, 1.00000E-01, 1.00000E-01, 1.00000E-01, 1.00000E-01, 1.00000E-01, 1.00000E-01, 1.00000E-01, 1.00000E-01, 1.00000E-01, 1.00000E-01, 1.00000E-01, 1.00000E-01, 1.00000E-01, 1.00000E-01, 1.00000E-01, 1.00000E-01, 1.00000E-01, 1.00000E-01, 1.00000E-01, 1.00000E-01, 1.00000E-01, 1.00000E-01, 1.00000E-01, 1.00000E-01, 1.00000E-01, 1.00000E-01, 1.00000E-01, 1.00000E-01, 1.00000E-01, 1.00000E-01, 1.00000E-01, 1.00000E-01, 1.00000E-01, 1.00000E-01, 1.00000E-01, 1.00000E-01, 1.00000E-01, 1.00000E-01, 1.00000E-01, 1.00000E-01, 1.00000E-01, 1.00000E-01, 1.00000E-01, 1.00000E-01, 1.00000E-01, 1.00000E-01, 1.00000E-01, 1.00000E-01, 1.00000E-01, 1.00000E-01, 1.00000E-01, 1.00000E-01, 1.00000E-01, 1.00000E-01, 1.00000E-01, 1.00000E-01, 1.00000E-01, 1.00000E-01, 1.00000E-01, 1.00000E-01, 1.00000E-01, 1.00000E-01, 1.00000E-01, 1.00000E-01, 1.00000E-01, 1.00000E-01, 1.00000E-01, 1.00000E-01, 1.00000E-01, 1.00000E-01, 1.00000E-01, 1.00000E-01, 1.00000E-01, 1.00000E-01, 1.00000E-01, 1.00000E-01, 1.00000E-01, 1.00000E-01, 1.00000E-01, 1.00000E-01, 1.00000E-01, 1.00000E-01, 1.00000E-01, 1.00000E-01, 1.00000E-01, 1.00000E-01, 1.00000E-01, 1.00000E-01, 1.00000E-01, 1.00000E-01, 1.00000E-01, 1.00000E-01, 1.00000E-01, 1.00000E-01, 1.00000E-01, 1.00000E-01, 1.00000E-01, 1.00000E-01, 1.00000E-01, 1.00000E-01, 1.00000E-01, 1.00000E-01, 1.00000E-01, 1.00000E-01, 1.00000E-01, 1.00000E-01, 1.00000E-01, 1.00000E-01, 1.00000E-01, 1.00000E-01, 1.00000E-01, 1.00000E-01, 1.00000E-01, 1.00000E-01, 1.00000E-01, 1.00000E-01, 1.00000E-01, 1.00000E-01, 1.00000E-01, 1.00000E-01), .Dim=c(25, 17)))*

*## OpenBUGS code ############################################################*

*# j is study and there are ns of these studies and m outcomes*

*# nt is the number of treatments*

*# ns is the number of studies*

*# type1[] is a vector containing a list of studies that are of type 1 eg. c(1,5,8,19)*

*# ns.type1 is the number of studies of type 1 (the length of the type1[] vector)*

*# r[j,k,i] are the number of events of type i in arm k of study j*

*# E[j,k] contains the exposure for study j, arm k*

*# E.observed[j,k] is 1 if E is observed*

*# na[j] are the number of arms in study j*

*# mu[j,i] is the baseline for study j for outcome type i*

*# t[j,k] is the treatment in arm k of study j*

*# d[t[j,k],i] is the log hazard ratio relative to treatment 1 for the treatment t[j,k] and outcome i*

*# R[j,k] is total number of events (of any type)*

*# n[j,k] are the number of patients in arm k of study j*

*# type2[] is a vector containing a list of studies that are of type 2 eg. c(2,3,4,9,13)*

*# f[j,k] contains mean follow-up time, if available, for study j and arm k and median otherwise*

*# f.observed[j,k] is 1 if the mean or median was observed in study j, arm k, 0 otherwise*

*# If the mean or median were not observed, f[j,k] contains the study duration*

*# may need to adapt to learn about pi across studies*

*# Includes five separate codings for the studies that report on 1st event data (type1)*

*# These are ACTIVE_W, AVERROES, BAFTA, PATAF, AFASAK II*

*# Outcome indicators are in active_w.outcomes, averroes.outcomes, bafta.outcomes,*

*# pataf.outcomes, and afasak_ii.outcomes*

*# Number of outcomes are n.active_w.outcomes, n.averroes.outcomes, n.bafta.outcomes,*

*# n.pataf.outcomes, and n.afasak_ii.outcomes*

*# Study indicators are active_w.j, averroes.j, bafta.j, pataf.j, afasak_ii.j*

*# Total number of events in each arm and of each event in particular are in active_w.R and active_w.r, etc.*

*model.af.competing.risks.fe<-function(){*

*for(j in 1:ns){*

*for(k in 1:na[j]){*

*# Impute the missing patient years at risk*

*E.completed[j,k] <- E.observed[j,k]*E[j,k] + (1-E.observed[j,k])*pi*E[j,k]*

*}*

*}*

*for(i in 1:m){*

*for(j in 1:ns){*

*mu[j,i] ~ dnorm(0, 0.5) # vague priors for trial baselines*

*# Model for the log hazards and log hazard ratios*

*for(k in 1:na[j]){*

*log(lambda[j,k,i]) <- mu[j,i] + d[t[j,k],i] - d[t[j,1],i]*

*}*

*}*

*d[1,i] <- 0 # treatment effect is zero for reference treatment*

*for(k in 2:nt){ d[k,i] ~ dnorm(0, 0.5) } # vague priors for treatment effects*

*}*

*#for(j in 1:ns.type1){*

*# for(k in 1:na[type1[j]]){*

*#sum.lambda[j,k]<-sum(lambda[type1[j],k,1:m]) # sum of the rates*

*# for(i in 1:m){*

*#lambda.scaled[j,k,i]<-lambda[type1[j],k,i]/sum.lambda[j,k] # scaled for multinomial likelihood*

*# }*

*# #R[type1[j],k]<-sum(r[type1[j],k,]) # Total number of events in arm k of study j*

*# R[type1[j],k] ~ dpois(theta1[type1[j],k])*

*# theta1[type1[j],k]<-E.completed[type1[j],k]*sum.lambda[j,k]*

*# r[type1[j],k,1:m] ~ dmulti(lambda.scaled[j,k,1:m],R[type1[j],k]) # Multinomial likelihood*

*# }*

*#}*

*# ACTIVE_W*

*for(k in 1:na[active_w.j]){*

*# Trial specific set of lambdas*

*for(i in 1:n.active_w.outcomes){*

*active_w.lambda[k,i]<-lambda[active_w.j,k,active_w.outcomes[i]]*

*}*

*sum.lambda[active_w.j,k]<-sum(active_w.lambda[k,])*

*# Divide the lambdas by their sum to scale them for the multinomial likelihood*

*for(i in 1:n.active_w.outcomes){*

*active_w.lambda.scaled[k,i]<-active_w.lambda[k,i]/sum.lambda[active_w.j,k]*

*}*

*active_w.R[k] ~ dpois(theta1[active_w.j,k])*

*theta1[active_w.j,k]<-E.completed[active_w.j,k]*sum.lambda[active_w.j,k]*

*# Multinomial likelihood*

*active_w.r[k,1:n.active_w.outcomes]~dmulti(active_w.lambda.scaled[k,1:n.active_w.outcomes],active_w.R[k])*

*}*

*# AVERROES*

*for(k in 1:na[averroes.j]){*

*# Trial specific set of lambdas*

*for(i in 1:n.averroes.outcomes){*

*averroes.lambda[k,i]<-lambda[averroes.j,k,averroes.outcomes[i]]*

*}*

*sum.lambda[averroes.j,k]<-sum(averroes.lambda[k,])*

*# Divide the lambdas by their sum to scale them for the multinomial likelihood*

*for(i in 1:n.averroes.outcomes){*

*averroes.lambda.scaled[k,i]<-averroes.lambda[k,i]/sum.lambda[averroes.j,k]*

*}*

*averroes.R[k] ~ dpois(theta1[averroes.j,k])*

*theta1[averroes.j,k]<-E.completed[averroes.j,k]*sum.lambda[averroes.j,k]*

*# Multinomial likelihood*

*averroes.r[k,1:n.averroes.outcomes]~dmulti(averroes.lambda.scaled[k,1:n.averroes.outcomes],averroes.R[k])*

*}*

*# BAFTA*

*for(k in 1:na[bafta.j]){*

*# Trial specific set of lambdas*

*for(i in 1:n.bafta.outcomes){*

*bafta.lambda[k,i]<-lambda[bafta.j,k,bafta.outcomes[i]]*

*}*

*sum.lambda[bafta.j,k]<-sum(bafta.lambda[k,])*

*# Divide the lambdas by their sum to scale them for the multinomial likelihood*

*for(i in 1:n.bafta.outcomes){*

*bafta.lambda.scaled[k,i]<-bafta.lambda[k,i]/sum.lambda[bafta.j,k]*

*}*

*bafta.R[k] ~ dpois(theta1[bafta.j,k])*

*theta1[bafta.j,k]<-E.completed[bafta.j,k]*sum.lambda[bafta.j,k]*

*# Multinomial likelihood*

*bafta.r[k,1:n.bafta.outcomes]~dmulti(bafta.lambda.scaled[k,1:n.bafta.outcomes],bafta.R[k])*

*}*

*# PATAF*

*for(k in 1:na[pataf.j]){*

*# Trial specific set of lambdas*

*for(i in 1:n.pataf.outcomes){*

*pataf.lambda[k,i]<-lambda[pataf.j,k,pataf.outcomes[i]]*

*}*

*sum.lambda[pataf.j,k]<-sum(pataf.lambda[k,])*

*# Divide the lambdas by their sum to scale them for the multinomial likelihood*

*for(i in 1:n.pataf.outcomes){*

*pataf.lambda.scaled[k,i]<-pataf.lambda[k,i]/sum.lambda[pataf.j,k]*

*}*

*pataf.R[k] ~ dpois(theta1[pataf.j,k])*

*theta1[pataf.j,k]<-E.completed[pataf.j,k]*sum.lambda[pataf.j,k]*

*# Multinomial likelihood*

*pataf.r[k,1:n.pataf.outcomes]~dmulti(pataf.lambda.scaled[k,1:n.pataf.outcomes],pataf.R[k])*

*}*

*# AFASAK II*

*for(k in 1:na[afasak_ii.j]){*

*# Trial specific set of lambdas*

*for(i in 1:n.afasak_ii.outcomes){*

*afasak_ii.lambda[k,i]<-lambda[afasak_ii.j,k,afasak_ii.outcomes[i]]*

*}*

*sum.lambda[afasak_ii.j,k]<-sum(afasak_ii.lambda[k,])*

*# Divide the lambdas by their sum to scale them for the multinomial likelihood*

*for(i in 1:n.afasak_ii.outcomes){*

*afasak_ii.lambda.scaled[k,i]<-afasak_ii.lambda[k,i]/sum.lambda[afasak_ii.j,k]*

*}*

*afasak_ii.R[k] ~ dpois(theta1[afasak_ii.j,k])*

*theta1[afasak_ii.j,k]<-E.completed[afasak_ii.j,k]*sum.lambda[afasak_ii.j,k]*

*# Multinomial likelihood*

*afasak_ii.r[k,1:n.afasak_ii.outcomes]~dmulti(afasak_ii.lambda.scaled[k,1:n.afasak_ii.outcomes],afasak_ii.R[k])*

*}*

*pi ~ dbeta(19,19) # Centered on 50% and approximately varying from 34% to 66%*

*for(j in 1:ns.type2) {*

*for(k in 1:na[type2[j]]){*

*# Impute the missing mean/median*

*f.completed[type2[j],k] <- f.observed[type2[j],k]*f[type2[j],k] + (1-f.observed[type2[j],k])*pi*f[type2[j],k]*

*r[type2[j],k,m] ~ dpois(theta.m[type2[j],k]) # Separate mortality likelihood*

*theta.m[type2[j],k]<- E.completed[type2[j],k]*lambda[type2[j],k,m]*

*for(i in 1:(m-1)){*

*r[type2[j],k,i] ~ dbin(p[type2[j],k,i],n[type2[j],k]) # Independent non-mortality events*

*cloglog(p[type2[j],k,i]) <- log(f.completed[type2[j],k]) + log(lambda[type2[j],k,i])*

*}*

*}*

*}*

*for(j in 1:ns.type3){*

*for(i in 1:m){*

*for(k in 1:na[type3[j]]){*

*r[type3[j],k,i] ~ dpois(theta3[type3[j],k,i])*

*theta3[type3[j],k,i] <- E.completed[type3[j],k] * lambda[type3[j],k,i]*

*}*

*}*

*}*

*# pairwise HRs and LHRs for all possible pair-wise comparisons*

*for(i in 1:m){*

*for (c in 1:(nt-1)) {*

*for (k in (c+1):nt) {*

*hr[c,k,i] <- exp(d[k,i] - d[c,i])*

*lhr[c,k,i] <- (d[k,i]-d[c,i])*

*}*

*}*

*}*

*# Only report outcomes of interest*

*for(i in 1:n.to.save)*

*{*

*for(k in 1:nt)*

*{*

*d.save[k,i]<-d[k,outcomes.to.save[i]]*

*}*

*}*

*}*
